# Are Small Nucleolar RNAs “CRISPRable”? A Report on Box C/D Small Nucleolar RNA Editing in Human Cells

**DOI:** 10.3389/fphar.2019.01246

**Published:** 2019-11-04

**Authors:** Julia A. Filippova, Anastasiya M. Matveeva, Evgenii S. Zhuravlev, Evgenia A. Balakhonova, Daria V. Prokhorova, Sergey J. Malanin, Raihan Shah Mahmud, Tatiana V. Grigoryeva, Ksenia S. Anufrieva, Dmitry V. Semenov, Valentin V. Vlassov, Grigory A. Stepanov

**Affiliations:** ^1^Institute of Chemical Biology and Fundamental Medicine, Siberian Branch of the Russian Academy of Sciences, Novosibirsk, Russia; ^2^Department of Natural Sciences, Novosibirsk State University, Novosibirsk, Russia; ^3^Institute of Fundamental Medicine and Biology, Kazan Federal University, Kazan, Russia; ^4^Department of Biological and Medical Physics, Moscow Institute of Physics and Technology (State University), Moscow, Russia; ^5^Laboratory of Cell Biology, Federal Research and Clinical Center of Physical-Chemical Medicine, Federal Medical Biological Agency, Moscow, Russia

**Keywords:** snoRNA, Gas5, genome editing, CRISPR/Cas9, box C/D snoRNA, RNA modification, alternative splicing, m^6^A

## Abstract

CRISPR technologies are nowadays widely used for targeted knockout of numerous protein-coding genes and for the study of various processes and metabolic pathways in human cells. Most attention in the genome editing field is now focused on the cleavage of protein-coding genes or genes encoding long non-coding RNAs (lncRNAs), while the studies on targeted knockout of intron-encoded regulatory RNAs are sparse. Small nucleolar RNAs (snoRNAs) present a class of non-coding RNAs encoded within the introns of various host genes and involved in post-transcriptional maturation of ribosomal RNAs (rRNAs) in eukaryotic cells. Box C/D snoRNAs direct 2’-O-methylation of rRNA nucleotides. These short RNAs have specific elements in their structure, namely, boxes C and D, and a target-recognizing region. Here, we present the study devoted to CRISPR/Cas9-mediated editing of box C/D snoRNA genes in *Gas5*. We obtained monoclonal cell lines carrying mutations in snoRNA genes and analyzed the levels of the mutant box C/D snoRNA as well as the 2’-O-methylation status of the target rRNA nucleotide in the obtained cells. Mutations in *SNORD75* in the obtained monoclonal cell line were shown to result in aberrant splicing of *Gas5* with exclusion of exons 3 to 5, which was confirmed by RT-PCR and RNA-Seq. The obtained results suggest that *SNORD75* contains an element for binding of some factors regulating maturation of Gas5 pre-lncRNA. We suggest that METTL3/METTL14 is among such factors, and m^6^A-methylation pathways are involved in regulation of *Gas5* splicing. Our results shell light on the role of *SNORDs* in regulating splicing of the host gene.

## Introduction

**Figure 1:**
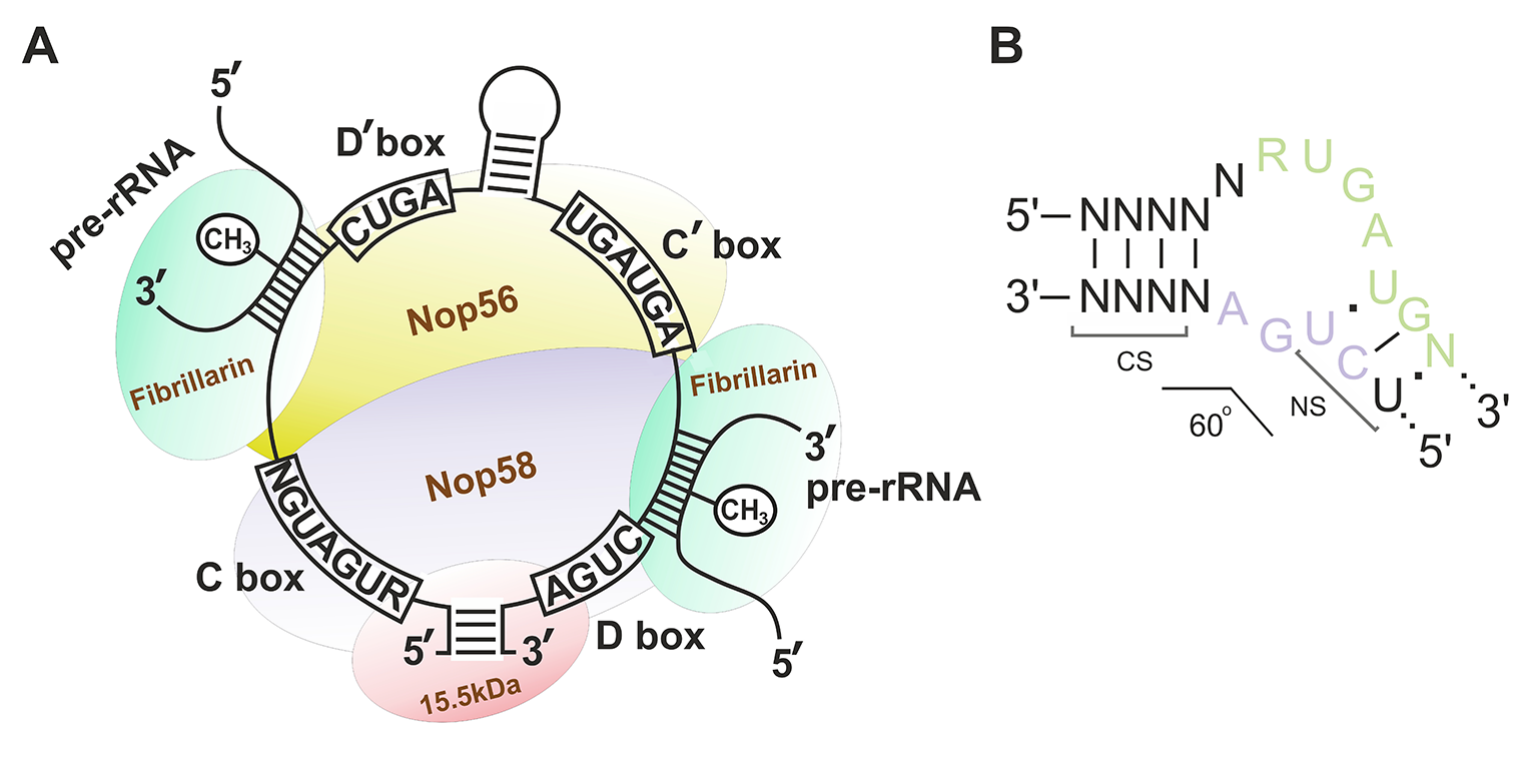
**(A**–**B)** Box C/D small nucleolar RNA structure. **(A)** The structure of the small nucleolar ribonucleoprotein (snoRNP) complex. Sequences of the conserved regions are indicated in capital letters. CH_3_ indicates the position of the nucleotide to be methylated in the target rRNA. Fibrillarin (methyltransferase), 15.5kDa, Nop56, and Nop58 are the small nucleolar proteins. **(B)** Secondary structure of the consensus Kink-turn motif in box C/D snoRNAs. CS, canonical stem; NS, non-canonical stem. R stands for purine; N denotes any nucleotide (mostly adenosine).

Box C/D snoRNAs present one of the two subclasses of small nucleolar RNAs (snoRNAs) responsible for post-transcriptional maturation of ribosomal RNAs (rRNAs) in eukaryotic cells: they guide 2’-O-methylation (2’-O-Me) of rRNA nucleotides (Kiss-László et al., 1996). Ribose methylation (2’-O-Me) is one of the most frequent types of nucleotide modification (alongside with pseudouridylation) in eukaryotic rRNA, with each of the 2’-O-Me sites being modified by a specific box C/D snoRNA (Cavaillé et al., 1996; Kiss, 2001; Piekna-Przybylska et al., 2007). Box C/D snoRNAs, in their turn, can have one or two targets: there are one or two 10–21 nucleotide guide sequences in the structure of snoRNA, which exhibit complementarity to a specific region within rRNA. There are also conserved elements, so-called boxes C and D, in the structure of these regulatory RNAs; these elements are required for the recognition of snoRNA-associated proteins followed by formation of the functionally active small nucleolar ribonucleoprotein (snoRNP) complexes (**Figure 1A**) (Terns and Terns, 2002; Reichow et al., 2007; Massenet et al., 2017). Terminal regions of a box C/D snoRNA molecule are complementary to each other; they, altogether with boxes C and D, form a stem-bulge-stem structure named “Kink-turn” (K-turn) (**Figure 1B**) (Watkins et al., 2000; Szewczak, 2005). The K-turn is recognized by core box C/D snoRNA proteins and required for proper processing, functioning, and localization of a mature snoRNP (Cavaillé and Bachellerie, 1996; Watkins et al., 1996; Škovapačková et al., 2010).

Apart from the canonical role of box C/D snoRNAs, several members of the class are known to perform other functions in the cell. According to the snoRNABase (www-snorna.biotoul.fr) (Lestrade, 2006), over a half of all human box C/D snoRNAs are orphan snoRNAs, since they have no identified 2’-O-methylation targets, while the real function in the cell remains unknown for most of them (Deschamps-Francoeur et al., 2014). However, for some of these snoRNAs, non-canonical functions have been elucidated. For instance, U3, U8, and U13 snoRNAs perform endonucleolytic cleavage of pre-rRNA (precursor of rRNA) and ensure correct folding of the resulting rRNA (Kass, 1990; Peculis and Steitz, 1993; Cavaille et al., 1996). SNORD115 (M/HBII-52) is involved in the regulation of the serotonin 2C receptor (5-HT2CR) mRNA level through alternative splicing and control of the target mRNA editing (Vitali et al., 2005; Kishore, 2006). SNORD115 and SNORD116 (M/HBII-85), both of which are encoded within the same locus (SNURF-SNRPN), are processed into smaller RNA forms, which, in their turn, are associated with splicing of various mRNA precursors (Kishore et al., 2010). A series of box C/D snoRNAs were shown to be further processed into miRNA-like derivatives, namely, snoRNA-derived RNAs (sdRNAs). Some of these snoRNA derivatives not only undergo Dicer-dependent processing pathway and associate with Ago family proteins (Ender et al., 2008; Burroughs et al., 2011) but also demonstrate miRNA activity (Brameier et al., 2011; Li et al., 2011; Patterson et al., 2017). Long RNA forms containing snoRNA in their structure (sno-lncRNAs) have been also detected in human cells (Yin et al., 2012). A novel function has been found for two orphan box C/D snoRNAs in yeasts: guiding of acetylation of two cytosine residues in 18S rRNA (Sharma et al., 2017). Recent papers also demonstrate evidence that individual snoRNAs guide 2’-O-methylation of tRNA (Vitali and Kiss, 2019) and mRNA (Elliott et al., 2019). A series of studies have also demonstrated the involvement of snoRNAs in such cellular processes as PKR activation (Youssef et al., 2015; Stepanov et al., 2018), cellular response to lipotoxicity (Michel et al., 2011; Holley et al., 2015), cholesterol trafficking (Brandis et al., 2013), and glucose metabolism (Lee et al., 2016).

One of the main protein components of a box C/D snoRNP is fibrillarin, which presents a 2’-O-methyltransferase (Tollervey et al., 1993; Kiss-László et al., 1996). Small nucleolar RNA species that do not perform ribose methylation are associated with different proteins than 2’-O-methylating ones. Their ribonucleoprotein complexes lack fibrillarin and other canonical snoRNP proteins (Falaleeva et al., 2016). Instead of that, these snoRNAs are a part of a spliceosome or associated with various RNA-binding proteins such as hnRNPs, ELAVL1, and RNA helicases (Tycowski et al., 1996; Soeno et al., 2010). Thus, the recent results indicate a vast variety of snoRNA roles as well as their structural forms that are found in the cells and required for implementation of their non-canonical functions.

Taking into account the specificity of the structure of box C/D snoRNAs and their target recognition ability, this class of regulatory RNAs presents a promising model for obtaining novel regulators of various processes, including post-transcriptional maturation. In addition, due to the fact that snoRNAs are encoded within the introns of various host genes, their knockout or mutation will not result in a frameshift and not necessarily lead to any drastic changes in the expression of the host gene, which is an another crucial aspect for selecting snoRNAs as a model in such studies. The aim of the study was to assess the possibility of selective editing of snoRNA genes in human cells using CRISPR/Cas9 tools.

## Materials and Methods

### Plasmids and Oligonucleotides

**Figure 2:**
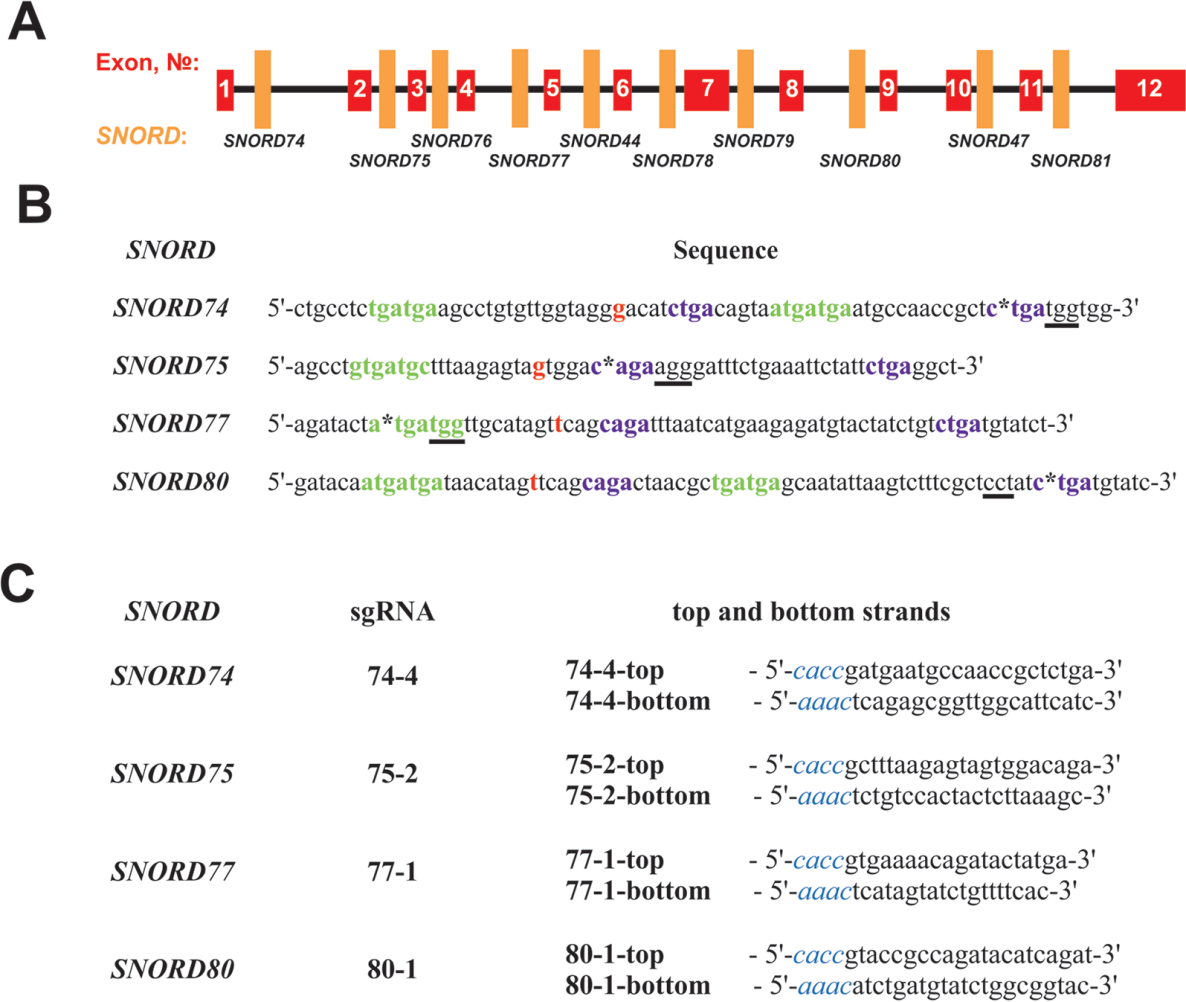
**(A**–**C)** Design of sgRNAs targeted at *Gas5* box C/D snoRNAs. **(A)**—Exon/intron structure of *Gas5* with positions of *SNORDs*. **(B)**—Sequences of the selected target snoRNAs with positions of PAM sequences (underlined) and CRISPR/Cas9 cleavage sites (asterisks). C and D boxes are indicated by green and purple font, respectively. Nucleotide complementary to the 2’-O-methylation site in the target rRNA is indicated in bold red. **(C)**—Sequences of the top and bottom strands of sgRNAs. Overhangs for ligation into the pair of BstV2I sites in pX458 are shown in blue italics.

A number of protospacer sequences were selected for specific cleavage of snoRNA genes encoded within *Gas5* (growth arrest-specific 5) introns. The protospacers were tested for possible off-target effects using Benchling tool (Benchling, RRID : SCR_013955). Plasmid pSpCas9(BB)-2A-GFP (pX458) (Addgene, #48138) was used as the expression vector (Ran et al., 2013). The corresponding oligonucleotides (“top and bottom strands” in **Figure 2C**) were annealed and cloned into the pX458 vector using BstV2I restriction endonuclease (SibEnzyme, Russia) and T4 DNA ligase (Thermo Fisher Scientific) according to (Ran et al., 2013). Competent TOP10 *Escherichia coli* cells were transformed with the obtained constructs, spread onto LB agar plates supplemented with ampicillin and incubated overnight at 37°C. Colonies containing pX458 plasmid with single guide RNA (sgRNA) insertion were selected by colony PCR and Sanger sequencing; CRISPR/Cas9 expression vectors were isolated using “EndoFree Plasmid Maxi Kit” (Qiagen).

### Cell Culture and Transfection

Human 293FT cell line (Thermo Fisher Scientific) was used in the study. Cells were cultured in DMEM/F12 medium containing 10% FBS and supplemented with 1x solutions of MEM NEAA, sodium pyruvate, GlutaMax, and Anti-Anti with addition of MycoZap Prophylactic (200 µl per 100 ml of medium) at 37°C and 5% CO_2_. All medium components, except for MycoZap (Lonza), were purchased from Gibco. Cells were seeded in six-well plates at a density of ∼0.3 x 10^6^ cells per well 24 h prior to transfection. Transfection of the cells with the expression vector was performed in RPMI medium using Lipofectamine 3000 Reagent (Thermo Fisher Scientific) according to the manufacturer’s instructions. Cells transfected with pX458 plasmid without sgRNA were used as the control.

### Single Clone Selection and Identification of Mutations

Cells were seeded at the amount of 1 cell per well in a 96-well plate by FACS (S3e Cell Sorter, Bio-Rad) 48 h after transfection. After reaching a ∼80–90% confluency, cells were divided into two equal portions and seeded in two 96-well plates. One of the plates was used for mutation screening by T7 endonuclease I (T7EI) cleavage assay (Kim et al., 2009). Genomic DNA was isolated using genomic DNA Isolation Kit (BIOLABMIX Ltd., Novosibirsk, Russia), PCR was performed using specific primers (5’-AGCCTTTGTCTGCTAAGGTCA-3’ and 5’-GTTGCCAT​TAACCGATGTCGA-3’ for *SNORD74*, 5’-TGGTATGTTACC​TGCATCATTGG-3’ and 5’-TAGGTGTACTCTCTATGTT​CCC-3’ for *SNORD75*, 5’-GAGTGCTAGAATGATGAGG-3’ and 5’-TCCAGCTTTCTGTCTAATGCC-3’ for *SNORD77*, 5’-ATTACAGGCATGTGACACC-3’ and 5’-CACTCCCA​TCTACAGATTAAGG-3’ for *SNORD80*), and the amplification products were annealed and subjected to T7EI (NEB) according to the manufacturer’s protocol. Mutations were identified by TA-cloning of the PCR products using CloneJET Kit (Thermo Fisher Scientific) and *E. coli* strain XL1-Blue followed by Sanger sequencing with further analysis of the obtained data by Tracking of Indels by Decomposition (TIDE) assay (Brinkman et al., 2014).

### Isolation of Total Cell RNA

Total RNA was isolated from control cells and clones using LIRA reagent (BIOLABMIX Ltd., Novosibirsk, Russia) according the manufacturer’s protocol and analyzed on a 1.5% agarose gel or using an Agilent 2100 Bioanalyzer.

### Differential Gene Expression Analysis

PolyA RNA fraction analysis with sequencing was performed using an Illumina NextSeq platform. Sequencing data (FASTQ formatted reads) were applied to the RNA-Seq workflow, which includes removing of adaptor-sequences with Trimmomatic V 0.38 (Bolger et al., 2014), mapping reads with HiSAT2 (Kim et al., 2015) on hg19, and transcriptome assembly with Cufflinks (NCBI RefSeq). The comparison of the expression levels of genes and transcripts in RNA-Seq experiments was carried out using CuffDiff (Trapnell et al., 2012). The list of differentially expressed genes (CuffDiff FDR adjusted after Benjamini–Hochberg correction of *p*-value for multiple-testing *q* < 0.05) was applied to the gene enrichment analysis powered by the Enrichr platform (Kuleshov et al., 2016). The RNA-Seq data have been deposited in ArrayExpress database under accession number E-MTAB-8269. Differential splicing analysis was performed using rMATS splicing tool as described (Anufrieva et al., 2018).

### Real-Time RT-PCR

Prior to RT-PCR, total RNA was isolated from the cells and treated with DNase I (Thermo Fisher Scientific). Quantitative RT-PCR was performed using BioMaster RT-PCR SYBR Blue reaction mix (BIOLABMIX Ltd., Novosibirsk, Russia) on a LightCycler 96 (Roche, Switzerland) with the following primers: U74: 5’-CTGCCTCTGATGAAGCCTGTG-3’ (U74-f) and 5’-CCACCATCAGAGCGGTTG-3’ (U74-r) or 5’-GAGCGG​TTGGCATTCATC-3’ (U74-all-r); U75: 5’-GTCGTATCCAGT​GCAGGGTCCGAGGTATTCGCACTGGATACGACAG​CCTC-3’ (U75-SL-sl-r), 5’-GTATACAGCCTGTGATG​CTTT-3’ (U75-SL-f), 5’-GTGCAGGGTCCGAGGT-3’ (U75-SL-r), and 5’-FAM-TGGATACGACAGCCTCAG-BHQ1-3’ (U75-SL-probe); U77: 5’-AGATACTATGATGGTTGC-3’ (U77-f) or 5’-ATGATGGTTGCATAGTTCAG-3’ (U77-all-f) and 5’-GA​TACATCAGACAGATAG-3’ (U77-r); U80: 5’-ACAATGATGA​TAACATAG-3’ (U80-f) and 5’-GATAGGAGCGAAAGACT-3’ (U80-all-r) or 5’-CATCAGATAGGAGCGAA-3’ (U80-r); Gas5: 5’-GAGGTAGGAGTCGACTCCTGTGA-3’ (exon 1 forward), 5’-GTGGAGTCCAACTTGCCTGGAC-3’ (exon 6 forward), 5’-CTGCATTTCTTCAATCATGAAT-3’ (exon 9 reverse); U1: 5’-CAGGGGAGATACCATGATCACGAAG-3’ and 5’-CGC​AGTCCCCCACTACCACAAAT-3’ U6: 5’-TCGCTTCGGCAG​CACATATACTAAAAT-3’ and 5’-GAATTTGCGTGTCATCCT​TGCG-3’ U8: 5’- AATCAGACAGGAGCAATCA-3’ and 5’-ATC​GTCAGGTGGGATAATCCT-3’ HPRT: 5’-CATCAAAGCACTG​AATAGAAAT-3’ and 5’-TATCTTCCACAATCAAGACATT-3’ B2M: 5’-CGCTCCGTGGCCTTAGCTGT-3’ _and 5’-AAAGA​CAAGTCTGAATGCTC-3’ 18S rRNA: 5’-GATGGTAGTC​GCCGTGCC-3’ and 5’-GCCTGCTGCCTTCCTTGG-3’ U47: TaqMan MicroRNA Assay #001223 (Thermo Fisher Scientific).

For assessment of the level of wild-type snoRNAs, the following primers were used: U74-f and U74-r (U74 RNA); U75-SL-sl-r, U75-SL-f, U75-SL-r and U75-SL-probe (U75 RNA); U77-f and U77-r (U77 RNA); and U80-f and U80-r (U80 RNA). For evaluation of the total level of all mutant RNA forms of the target snoRNA in the corresponding clone, the following primers were used: U74-f and U74-all-r (U74 RNA forms); U75-SL-sl-r, U75-SL-f and U75-SL-r (U75 RNA forms); U77-all-f and U77-r (U77 RNA forms); and U80-all-r and U80-f (U80 RNA forms).

The expression of target genes is presented as values normalized to the endogenous level of 18S rRNA, *HPRT, B2M* mRNA, U1, U6, U8, and U47 RNA. The mean values [ ± standard deviation (SD)] from three independent experiments were represented.

### Analysis of the Relative Level of 2’-O-Me of the Target rRNA Nucleotide

#### Partial Alkaline Hydrolysis

Partial alkaline hydrolysis of RNAs was performed as described in (Kiss-László et al., 1996). Reverse transcription was performed using primers containing a 5’-terminal [^32^P] label: 5’-CGTTCCCTTGGCTGTGGT-3’ (C3820 28S rRNA, U74 RNA), 5’-GCCTCACCGGGTCAGTGA-3’ (C4032 28S rRNA, U75 RNA), and 5’-GTCAGGACCGCTACGGACCTC-3’ (A1521 28S rRNA, U77/U80 RNA). Sequencing of the region of 28S rRNA was performed as described in (Filippova et al., 2015).

#### RT-PCR-Based Method

Reverse transcription followed by PCR with modification-specific primers was performed using total RNA samples isolated from the control and monoclonal cells. The following primer sets were used for analysis of the methylation status of C3820 28S rRNA, C4032 28S rRNA, and A1521 28S rRNA, respectively: forward 5’-GAACGAGATTCCCACTG-3,’ reverse 5’-CCGTTC​CCTTGGTGTG-3,’ inside primer 5’-GATTCCCACTGTC​CCTACC-3’; forward 5’-CCGCCGGTGAAATACCA-3,’ reverse 5’-AACTCCCCACCTGGCACT-3,’ inside primer 5’-GAA​ATACCACTACTCTGATCG-3’; and forward 5’-AGGACCCGA​AAGATGGTGA-3,’ reverse 5’-GTCAGGACCGCTACGGAC​CTC-3,’ inside primer 5’-AAGATGGTGAACTATGCCTG-3.’ For each of the samples, reactions with 1.0 mM (or 1.5 mM) and 3.0 mM (or 0.01 mM) dNTP concentrations were performed in parallel. Relative change in the modification level of the target nucleotide was evaluated based on the difference between the amplification level for the study and control samples at suboptimal dNTP concentration. The approach is based on the method of identification of 2’-O-Me groups in rRNA by RT-PCR first presented by Belin et al. (2009).

#### RNase H- and HPLC-MS/MS-Based Method

For analysis of the 2’-O-methylation status by HPLC-MS/MS, rRNA was separated from short RNA forms using miRNA isolation kit LRU-100-50 (BIOLABMIX Ltd., Novosibirsk, Russia). A total of 3 µg of rRNA were incubated with 1 µM oligonucleotides in a buffer containing 20 mM Tris-HCl, 40 mM KCl, 8 mM MgCl_2_, and 1 mM DTT (pH 7.8) at 37 °C for 30 min. The following pair of oligonucleotides was used: 5’-CACTTATTCTACACACCTC-3’ and 5’-CTCCCCCCACGGCACTGTC-3’

Next, RNase H (Thermo Scientific, USA) was added to the reaction mixture to a final concentration of 80 U/ml and incubated at 37 °C for 2 h. The RNase H cleavage products were precipitated in 2% LiClO4/acetone and then separated on a denaturing Page gel (**Supplementary Table 1**). The fragment of interest was subjected to enzymatic hydrolysis to nucleosides (Chan et al., 2010). High performance liquid chromatography coupled with tandem mass spectrometry (HPLC-MS/MS) was performed for quantitative assessment of the 2’-O-methylation status of the target nucleotide as described earlier (Stepanov et al., 2018).

### Real-Time Cell Proliferation Analysis

The viability and the number of cells were evaluated on the automated cell counter LUNA-II (Logos Biosystems, South Korea) using Trypan Blue Dye (Bio-Rad Laboratories). Cell proliferation was assessed by real-time cell analysis using electrical impedance assay—xCELLigence System (ACEA Bioscience, San Diego, CA, USA). RTCA software was used to determine CI values through the measured impedance recordings. Briefly, cells were plated in 16-well E-plates (ACEA Bioscience) at a density of 20,000 cells per well in a total volume of 200 µl of complete medium and were monitored in real-time mode. The data were recorded every hour for 62 h; cell indexes were calculated using RTCA Software 1.2 (ACEA Bioscience). Cell index is a parameter reflecting the impedance of electron flow caused by adherent cells.

### Statistical Analysis

Unpaired Student’s t-test was used to confirm the statistical significance of the differences (data are presented as means ± SD). The differences were considered statistically significant at *p*-value < 0.05 (*), *p*-value < 0.01 (**), and *p*-value < 0.001 (***).

## Results

### Design of sgRNAs Targeted at snoRNAs

We selected *Gas5* (*growth arrest-specific 5*) as the target gene, since it presents a well-studied multi-small-nucleolar-RNA host gene. A total of 10 box C/D snoRNAs are encoded within the introns of *Gas5*: *SNORD74*, *SNORD75*, *SNORD76*, *SNORD77*, *SNORD44*, *SNORD78*, *SNORD79*, *SNORD80*, *SNORD47*, and *SNORD81* (**Figure 2A**) (Smith and Steitz, 1998). Analysis of the genomic sequences of *Gas5* snoRNAs demonstrated the presence of protospacer adjacent motifs (PAMs, 5’-NGG-3’) in all of these box C/D snoRNAs. However, not all of the snoRNAs contain PAM sequences in the vicinity of the key functional elements, namely, boxes C, C,’ D, and D’ (**Figure 2**). The desired PAM positions were those located adjacent or within the conserved elements, since these regions are responsible for the recruitment of snoRNA-specific proteins and formation of the functionally active snoRNP complex. Even point mutations at specific positions, especially within the structure of functional elements, are known to abrogate snoRNA function (Cavaillé et al., 1996).

*SNORD74* and *SNORD75* contain more than one PAM in their structure (**Supplementary Table 3**). Of these, we have selected the cleavage sites located within the regions of boxes D and D’ in *SNORD74* and *SNORD75*, respectively (**Figure 2B**). *SNORD77* and *SNORD80*, on the contrary, contain only one PAM site, which is located within the regions of boxes C and D, respectively (**Figure 2B**, **Supplementary Table 3**). Four sgRNAs were constructed, with each of them targeting cleavage of either *SNORD74*, *SNORD75,* *SNORD77*, or *SNORD80*: 74-4, 75-2, 77-1, and 80-1, respectively (**Figure 2C**). Plasmids expressing the designed sgRNAs were obtained using the oligonucleotides presented in **Figure 2C**.

### CRISPR-Mediated Mutations in Conserved Elements Resulted in Downregulation of the Target snoRNAs

**Figure 3:**
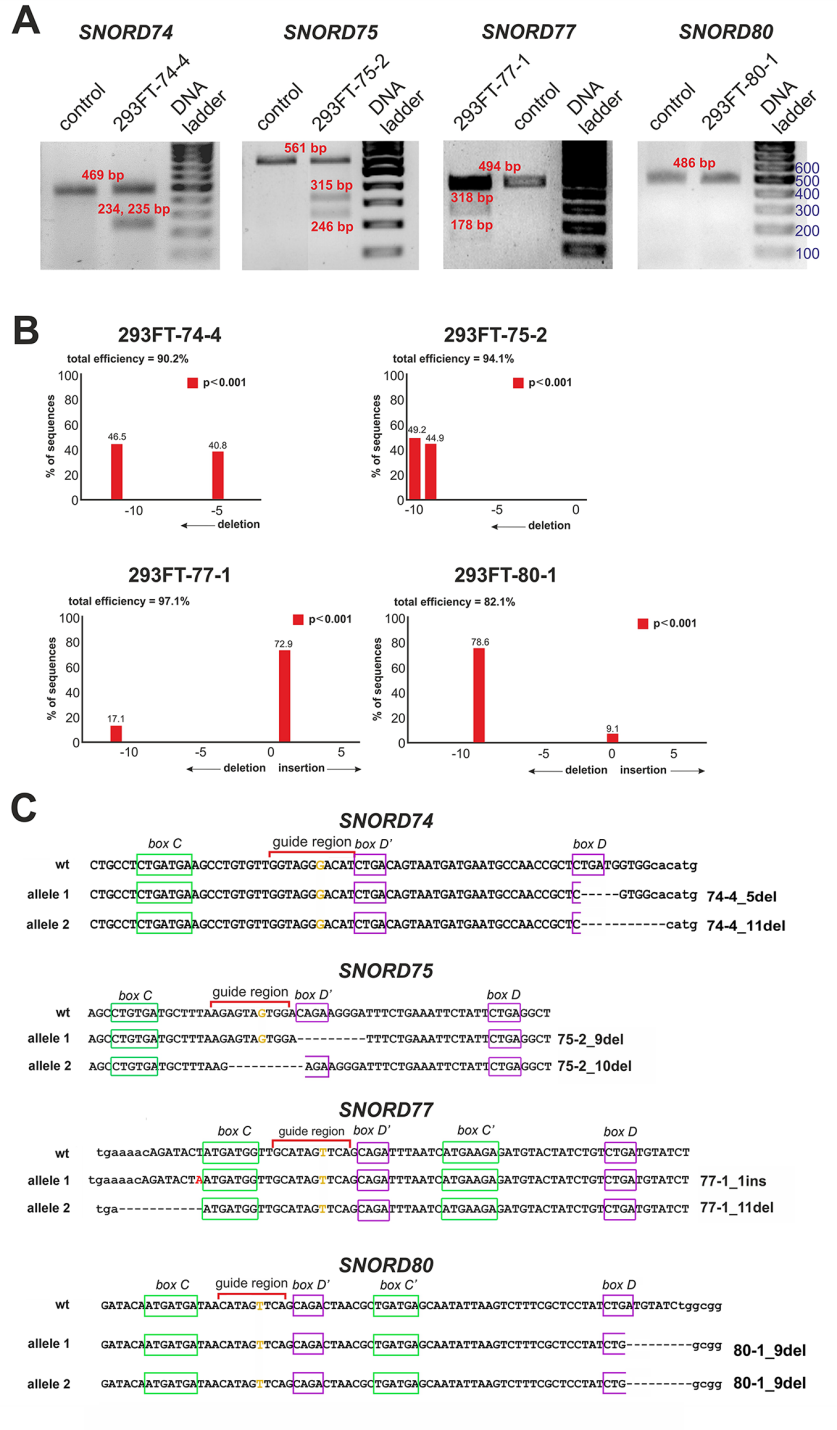
**(A**–**C)** CRISPR/Cas9-mediated mutations in the *Gas5* *SNORD*s in the obtained monoclonal cell lines. **(A)** T7 Endonuclease I assay with specific primers. Lengths of the Endonuclease restriction products correspond to the position of the CRISPR/Cas9 cleavage site in the target *SNORD*. **(B)** Sequencing of the genomic region of the target *SNORD* in the obtained cell lines followed by TIDE analysis. Position on the X axis indicates the number of deleted (negative value) or inserted (positive value) nucleotides, while Y value shows percentage distribution of the detected mutations. **(C)** Mutations in the alleles of the target *SNORD* in the obtained clones. Genomic sequences of the target snoRNAs are shown in capital letters. Conserved elements are framed in green and purple. The nucleotide denoted in ochre yellow in the guide region is complementary to the 2’-O-methylation site in the target rRNA. Insertion of 1 nucleotide in *SNORD77* is denoted by red font. Deletion is indicated by dash line. WT, wild type allele.

After transfection of 293FT cells with the obtained plasmids, they were sorted for GFP-positive cells, and viable monoclonal lines carrying mutations in the target snoRNA-encoding genes were selected. T7 endonuclease I assay demonstrated the presence of mutations in *SNORD74* (293FT-74-4 line), *SNORD75* (293FT-75-2 line), and *SNORD77* (293FT-77-1 line) (**Figure 3A**).

Further Sanger sequencing followed by TIDE assay revealed the presence of a 5-nt and a 11-nt deletions (74-4_5del and 74-4_11del, respectively) in *SNORD74* for clone 293FT-74-4 (**Figure 3B**). TA-cloning with sequencing of individual alleles demonstrated that both mutations partially covered the D box region as well as the 3’-terminal region involved in the Kink-turn formation (**Figure 3C**) (Watkins et al., 2000; Szewczak, 2005). A 9-nt and a 10-nt deletions (75-2_9del and 75-2_10del, respectively) were identified for *SNORD75* in the clone 293FT-75-2 (Figures 3B, C). These deletions covered partially (75-2_10del) or entirely (75-2_9del) the D’ box region. Clone 293FT-77-1 was shown to carry a 1-nt insertion (77-1_1ins) and a deletion of 11 nucleotides (77-1_11del) in the region adjacent to the C box sequence in *SNORD77* (**Figures 3B, C**).

Initially, no endonuclease I digestion products were obtained for the clone 293FT-80-1 (**Figure 3A**). We suggested that this is due to the presence of identical mutations in both alleles of *SNORD80*, since downregulation of the target snoRNA was observed in the obtained monoclonal cells. Indeed, TA-cloning with Sanger sequencing and analysis using TIDE software revealed a 9-nt deletion in the D box terminal region of the two alleles of *SNORD80* (**Figure 3B**). The mutation partially covered the D box and the 3’-terminal regions (**Figure 3C**).

Thus, four monoclonal cell lines were obtained, with each of them carrying mutations in both alleles of one of the target snoRNA genes: *SNORD74*, *SNORD75*, *SNORD77*, and *SNORD80*.

**Figure 4:**
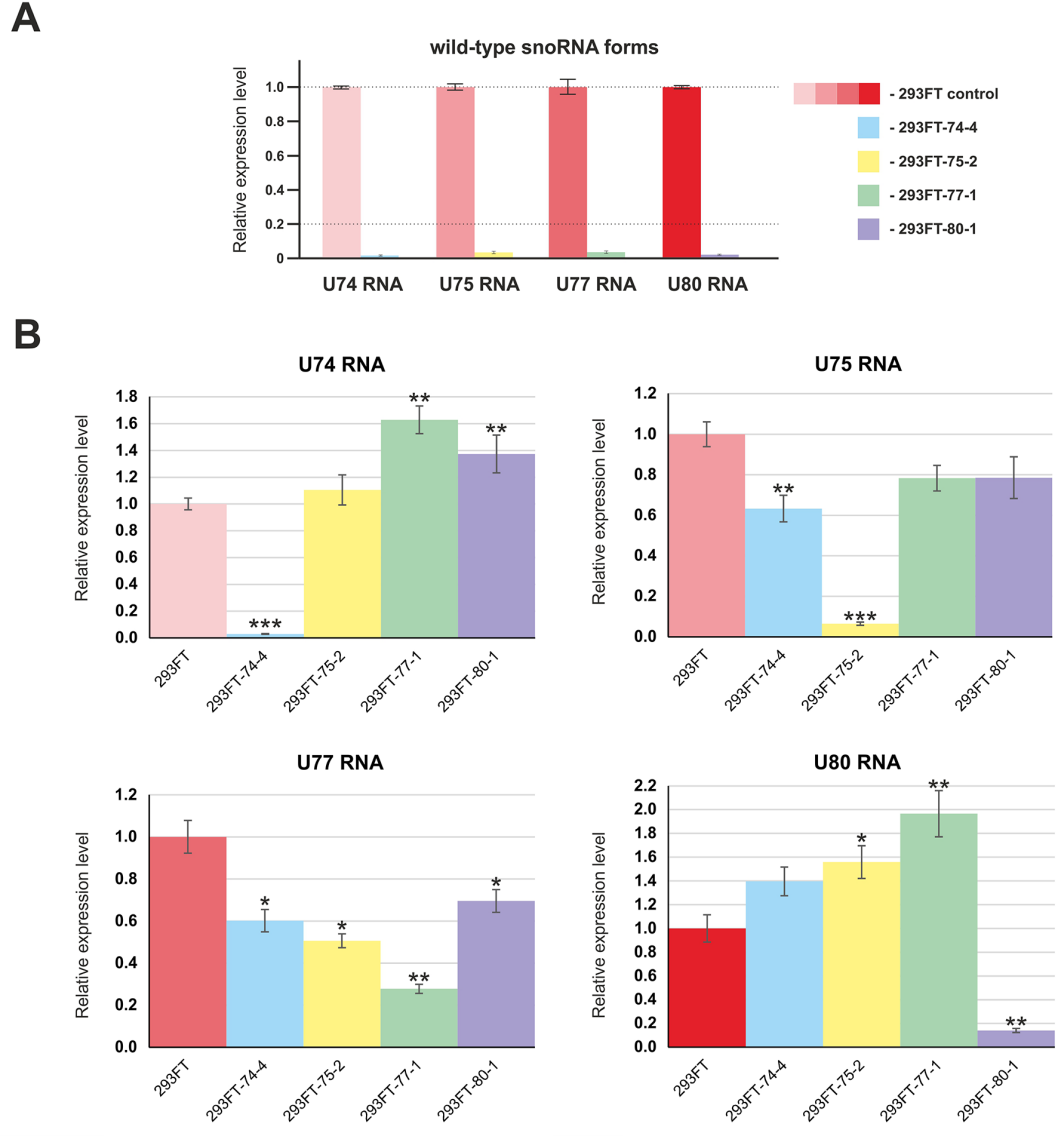
**(A**–**B)** Relative expression level of the target snoRNAs in the corresponding clones 293FT-74-4, 293FT-75-2, 293FT-77-1, and 293FT-80-1 compared to the 293FT cells evaluated by qRT-PCR with primers specific to wild-type **(A)** and mutant **(B)** snoRNA forms. The level of intact snoRNAs in each of the clone was evaluated using primers specific to wild-type snoRNA form **(B)**. All data are presented as mean ± SD. *p < 0.05 *vs*. control, **p < 0.01 *vs*. control, ***p < 0.001 *vs*. control.

First, we confirmed the absence of wild-type forms of the four target snoRNAs in the corresponding monoclonal cells by qRT-PCR (**Figure 4A**). Next, in order to evaluate the level of the target snoRNA in the corresponding clone, we used primers that allow detection of the mutant snoRNA forms. Real-time RT-PCR analysis demonstrated a decrease in the total level of each of the four snoRNAs in the corresponding monoclones. For instance, mutant *SNORD77* was expressed at 25% of the wild-type level, while mutant U74 RNA was not detected by RT-PCR in 293FT-74-4 (**Figure 4B**). The level of other *Gas5 SNORDs* also changed but not as dramatically as that for the target *SNORDs* (**Figure 4B**). The most significant changes were observed for *SNORD74* in 293FT-77-1 (1.6-fold increase compared the level of intact *SNORD74* in the control cells), *SNORD77* RNA in 293FT-75-2 (1.5-fold decrease), and *SNORD80* RNA in 293FT-75-2 (1.5-fold increase) and 293FT-77-1 (two-fold increase) cells.

We analyzed the nature of mutations in order to determine whether they can provide functional snoRNA forms that can further form snoRNP complexes and guide 2’-O-methylation of their target nucleotides. Since mutations in most of the obtained monoclones (except for *SNORD75* in 293FT-75-2) cover terminal conserved elements (**Figure 3C**), they might prevent formation of the proper K-turn structure. It is known that association of the snoRNA with the core snoRNP proteins, especially 15.5 kDa, is impossible in case if an aberrant C/D-motif is formed (Watkins et al., 2002).

**Figure 5:**
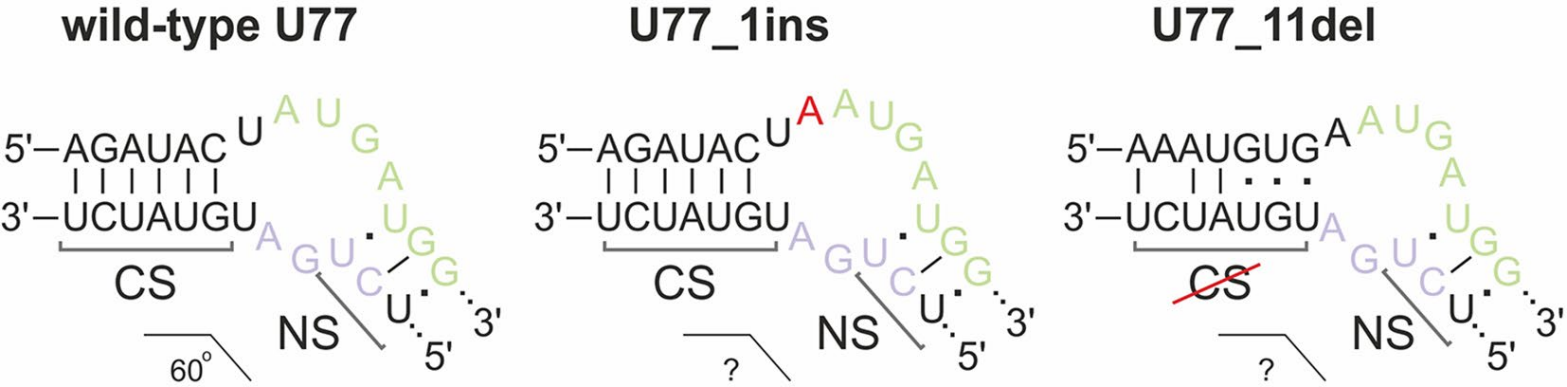
Kink-turn motif structure for wild-type U77 and potential structures for U77 mutants in 293FT-77-1. Insertion of 1 nucleotide in U77_1ins is denoted by red “A.” CS, canonical stem; NS, non-canonical stem.

We concluded that, of all of the target *SNORDs*, only *SNORD77* in 293FT-77-1 might provide functional snoRNA forms. Mutations in the alleles of *SNORD77* differ significantly: there is a 11-nt deletion of the 5’-terminal area in one of the alleles (77-1_11del) and a 1-nt insertion in the C-box-adjacent area (**Figure 3C**). Deletion of such a vast terminal region of 11 nt in 77-1_11del seems deleterious for formation of a proper canonical stem structure. We tried to estimate whether the region could be substituted with the adjacent intronic sequence in the mature RNA form (**Figure 5**). However, this sequence does not provide enough complementarity to form a canonical stem of the K-turn (Chagot et al., 2019). On the opposite, addition of one nucleotide (adenosine) beyond the functionally important region might not have such a tremendous effect, and U77_1ins might still provide a functional snoRNA (**Figure 5**).

However, the overall downregulation of *SNORD77* in the monoclone 293FT-77-1 indicates that the addition of one nucleotide in the K-turn area changes the spatial structure of the snoRNA and affects its interaction with snoRNA-associated proteins thus resulting in its dysfunction. The mutation carried in 77-1_1ins allele might affect the spatial parameters such as the angle between the two stems (canonical and non-canonical stem) within the K-turn structure and impair the snoRNA stability.

Mutations in *SNORD75* cover such essential elements as the D’ box area (75-2_9del) and the guide region (75-2_10del) (**Figure 3C**). Despite of the partial or complete deletion of the D’ box in 75-2_10del and 75-2_9del, respectively, the element can be substituted with a D/D’-box-resembling sequence (CUGA), which is located in the structure between the boxes D and D’ in wild-type U75. However, both mutations result in a significant shortening of U75 sequence sequence, as well as the distance between the boxes C and D’ and boxes D and D,’ and these parameters are known to be crucial for snoRNA functioning (Qu et al., 2011). Thus, it is unlikely that, if the mutant forms are somehow processed, the resulting snoRNA is not functional.

CRISPR/Cas9 cleavage of *SNORD74* and *SNORD80* resulted in impairment of the D box region (**Figure 3C**). Analysis of the structure of the mutant forms for these snoRNAs demonstrated that these variants cannot form a proper K-turn structure (**Supplementary Table 1** **Figure 1**).

**Figure 6:**
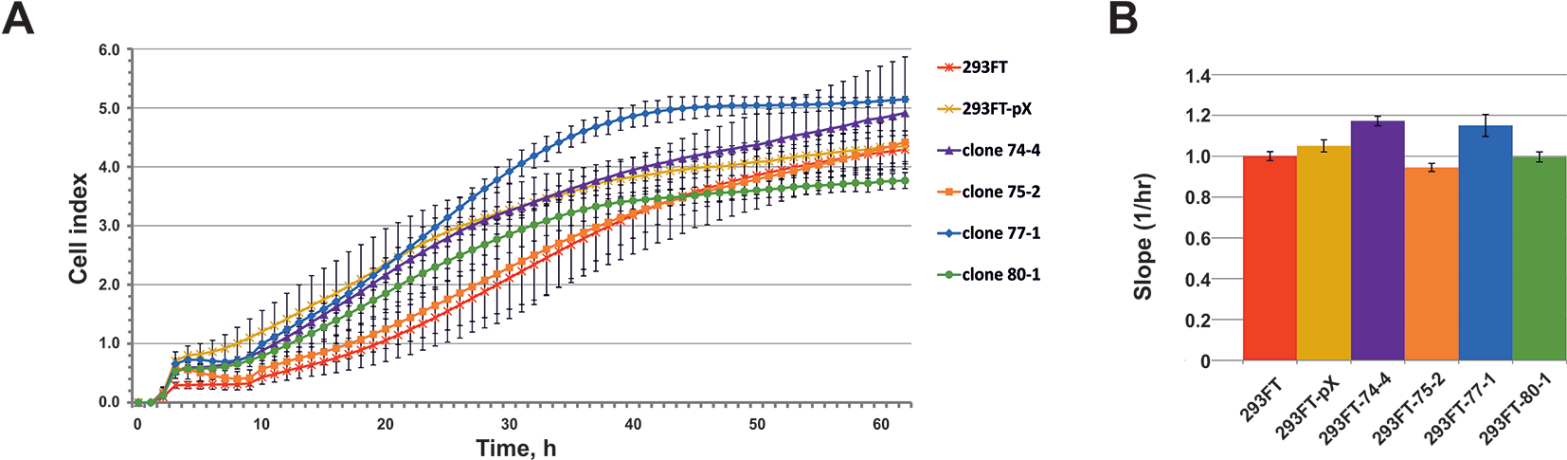
**(A**–**B)** Cell proliferation of the monoclones and control 293FT and 293FT-pX cells. **(A)** Representative cell index (mean ± standard deviation) as a measure of cell proliferation is depicted for clones 293FT-75-2 and 293FT-77-1, 293FT-74-4, and 293FT-80-1 and control 293FT and 293FT-pX cells. Measurements were automatically collected by the xCELLigence RTCA analyzer every 1 h for a period of 62 h (n = 2). **(B)** Results of the cell proliferation assay represented as slopes (changes in cell index/hour). Cells not carrying any plasmid (293FT) and cells transfected with pX458 plasmid without sgRNA (293FT-pX) were used as independent controls.

No significant differences in the growth rate were observed for all of the obtained clones compared to the control 293FT cells (**Figure 6**). 293FT-74-4 and 293FT-77-1 clones were characterized by insignificantly divergent growth rates compared to 293FT cells (**Figure 6B**). Functional analysis of RNA-Seq profiling of gene expression in obtained monoclonal and control cells did not reveal any significant activation of cell death pathways (**Supplementary Table 4**). The obtained results demonstrate that CRISPR/Cas9-mediated cleavage of snoRNA genes does not affect essential cellular processes and therefore can be used for obtaining stable cells expressing mutant snoRNAs.

### CRISPR-Mediated Mutations in snoRNAs Affect 2’-O-Methylation of rRNA

Since mutations in the structure of box C/D snoRNAs can lead to the loss of their functional ability to guide 2’-O-methylation, the target rRNA nucleotide might not contain the modification. In order to test this hypothesis, we analyzed the level of ribose methylation of the target sites in 28S rRNA for each of the snoRNA in the corresponding clones using the approach proposed by Belin et al. (2009).

The method is based on termination of reverse transcriptase enzyme at 2’-O-methylated sites in the template RNA at low dNTP concentrations (Maden et al., 1995; Maden, 2001). In some cases, the use of high dNTP concentrations instead of decreased concentrations might yield higher specificity and more accurate results (Filippova et al., 2015). Two reactions of reverse transcription of the total RNA sample are performed in parallel: at optimal (1.0 or 1.5 mM) and suboptimal (3.0 or 0.01 mM) dNTP concentrations (Filippova et al., 2017). The length of the reverse transcription product corresponds to the position of the 2’-O-Me in the template RNA. Therefore, the full-length cDNA product is amplified when using primers flanking the site of interest in case if the site is not modified while no amplification product is observed in case of truncated cDNA (in the presence of 2’-O-Me). Thus, the 2’-O-methylation level of the target site reversely correlates with the level of the PCR product.

**Figure 7:**
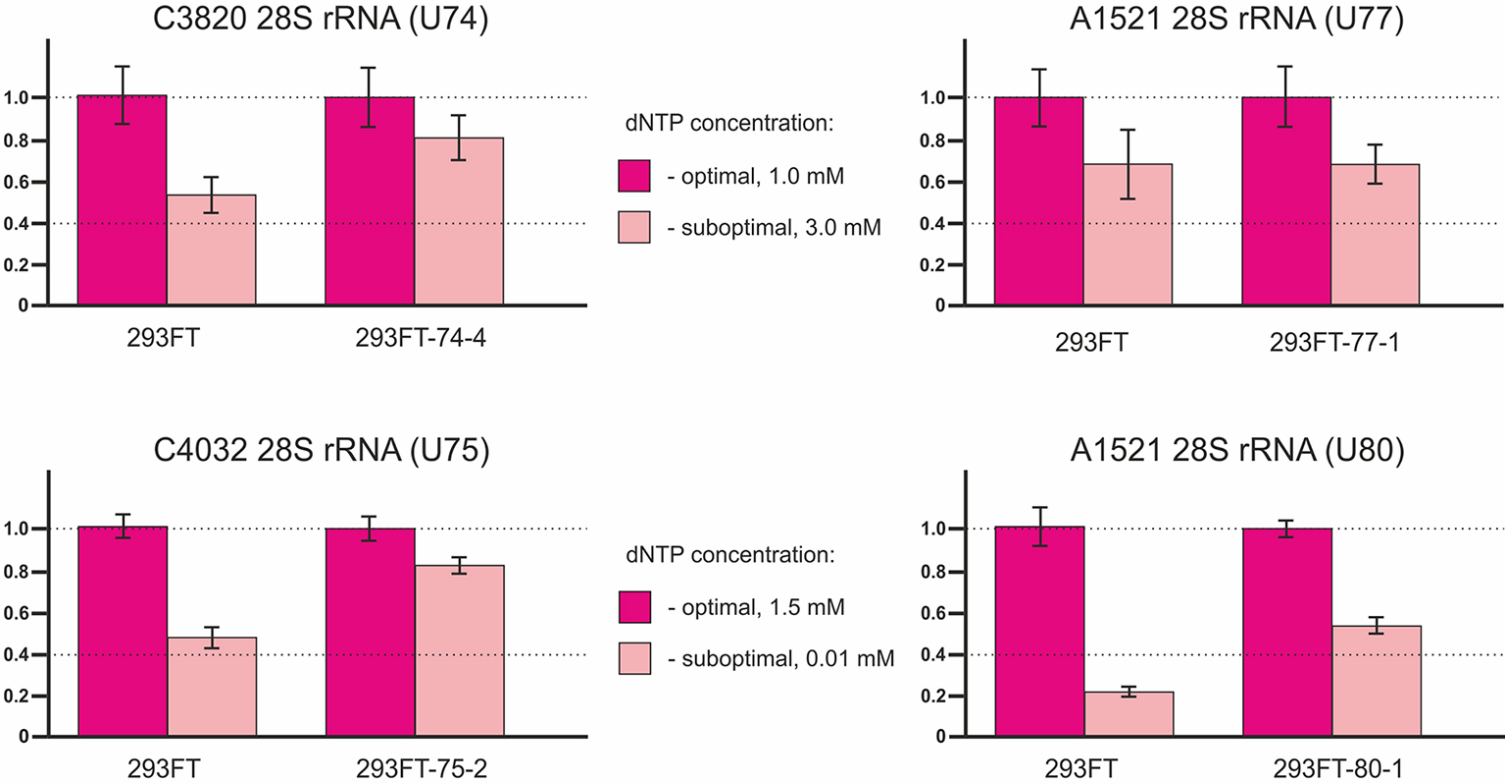
Relative changes in the 2’-O-methylation level of the target rRNA nucleotides for U74, U75, U77, and U80 RNAs in the clones 293FT-74-4, 293FT-75-2, 293FT-77-1, and 293FT-80-1, respectively, compared to the control 293FT cells evaluated by the method of reverse transcription termination followed by PCR.

The method of RT termination followed by PCR (Aschenbrenner and Marx, 2016) revealed a decrease in the 2’-O-methylation level of C3820 (∼34% decrease), C4032 (∼42% decrease), and A1521 (∼60% decrease) 28S rRNA in 293FT-74-4, 293FT-75-2, and 293FT-80-1 monoclones, respectively, compared to the control cells (**Figure 7**). The only monoclone that demonstrated no changes in the 2’-O-Me level of the target rRNA site (A1521) was 293FT-77-1.

To confirm incomplete abrogation of the target site modification, we used independent approaches. The method of RNase H treatment followed by HPLC-MS/MS was used to verify the data on the 2’-O-methylation status of C4032 28S rRNA in the clone 293FT-75-2: a decrease in the 2’-O-methylation level was shown (**Supplementary Table 1** **Figures 2A, B**). However, the method of partial alkaline hydrolysis demonstrated that the modification was not abrogated completely (**Supplementary Table 1** **Figures 2C**).

Of special interest was to analyze the 2’-O-methylation status of the target rRNA nucleotide for U77 and U80 RNAs in the corresponding clones, since both snoRNAs share the same target, namely, A1521 28S rRNA. While RT-PCR-based method showed a decreased 2’-O-methylation level of the target nucleotide in 293FT-80-1, no changes were observed for 293FT-77-1 cells (**Figure 6**). The absence of changes for A1521 28S rRNA in 293FT-77-1 was confirmed by a modified RT-based approach, which has been developed by us earlier (Filippova et al., 2015) (**Supplementary Table 1** **Figure 3A**, **Supplementary Table 2**). Partial alkaline hydrolysis demonstrated that mutations in U77 and U80 RNAs do not abrogate 2’-O-methylation of the target nucleotide completely (**Supplementary Table 1** **Figure 3B**).

### CRISPR/Cas9-Mediated Cleavage of *Gas5* snoRNA Results in Downregulation of the Host Gene and Formation of an Alternative Splicing Variant

**Figure 8:**
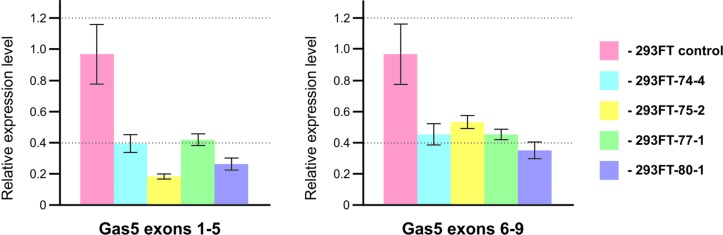
Level of the host-gene Gas5 lncRNA in the obtained clones. Real-time RT-PCR data on the level of Gas5 lncRNA with sets of primers complementary to exons 1, 5 and 6, 9.

In order to study the effects caused by CRISPR/Cas9-mediated mutations in the genes encoding snoRNAs on the level and maturation of the host gene lncRNA Gas5, we performed qRT-PCR analysis with the sets of primers complementary to various exons of *Gas5*. As a result, a decrease in the level of Gas5 RNA was shown for all of the obtained clones (**Figure 8**). It should be noted that the level of each of the studied *Gas5* snoRNAs altered independently of the others in the obtained monoclones (**Figure 4B**), which indicates the existence of an individual mechanism for regulation of the level of each of the *Gas5* snoRNAs.

**Figure 9:**
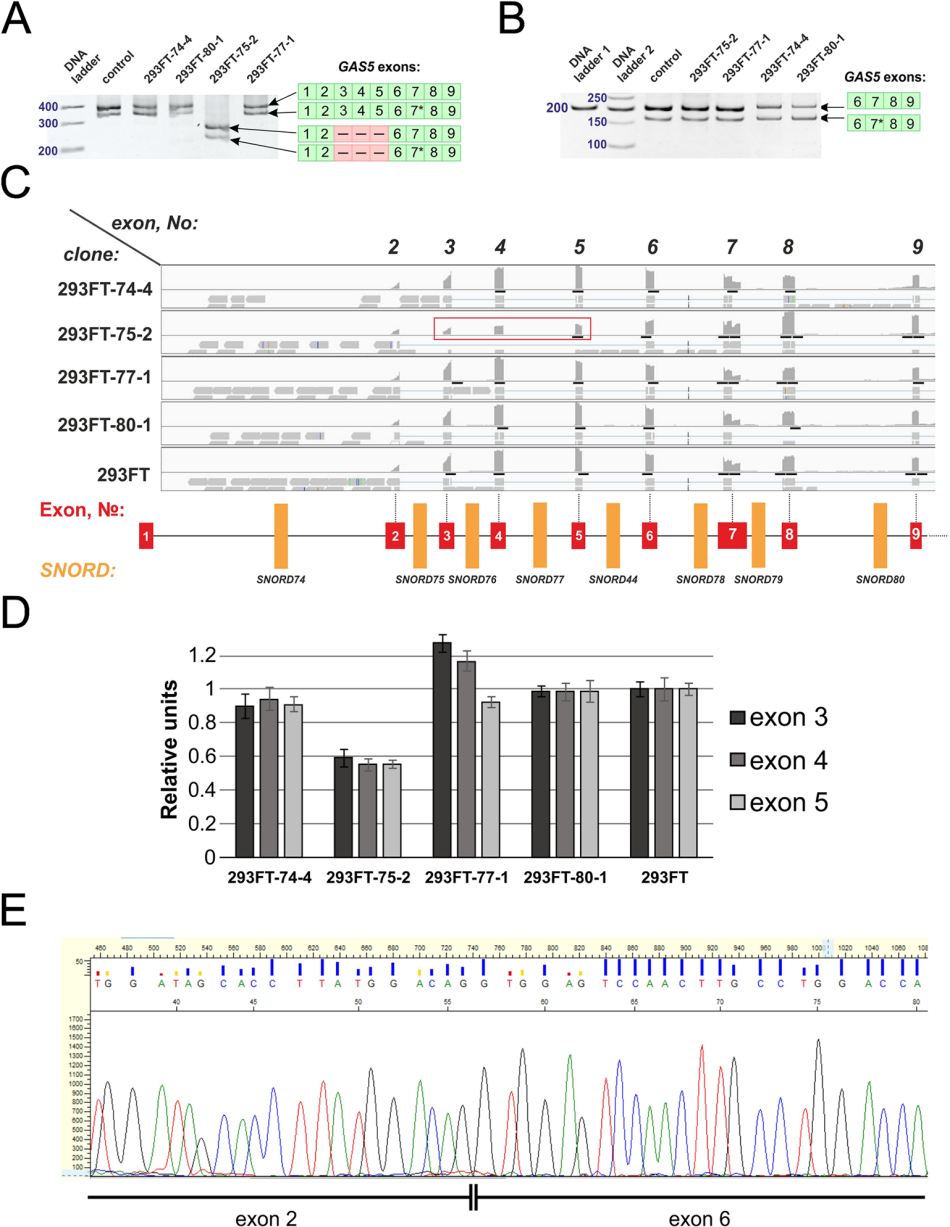
**(A**–**E)** Products of real-time RT-PCR from total cell RNA isolated from the clones and control 293FT cells with primers complementary to exons: 1–9 **(A)** and 6–9 **(B)**. Products were separated by 8% **(A)** and 5% **(B)** PAGE and stained with ethidium bromide. Figures in green squares indicate the numerical order of exons comprising the alternative transcript. Excised exons in the splicing variant detected in clone 293FT-75-2 are indicated by pink squares with dashed lines. 7*—Alternative splicing variant of Gas5 RNA containing a truncated exon 7 region. **(C)** RNA-Seq data representing the number of reads for exons 2 to 9 of *Gas5* in the clones and control cells. Exons 3, 4, and 5 that are absent in the novel alternative transcript in the clone 293FT-75-2 are encircled by red rectangle. **(D)** Graphs representing comparison of the relative number of reads (expressed as % of the maximum number of reads) for exons 3, 4, and 5 in each of the obtained monoclones and control cells. **(E)** Sanger sequencing of the alternative transcript lacking exons 3 to 5 in 293FT-75-2 monoclone.

We also observed the presence of a truncated splicing variant lacking some of the exons in 293FT-75-2 (**Figure 9A**). Two transcript variants of Gas5 lncRNA are detected in each of the obtained monoclones, as well as in control 293FT cells (**Figure 9A**). Both of these forms present naturally occurring transcript variants (NR_152525.1, 660 nt; NR_152530.1, 621 nt), which differ in the length of exon 7: the shorter transcript contains a truncated exon 7 region (**Figure 9B**). RNA-Seq data and Sanger sequencing of the alternative variant product revealed the presence of a mature Gas5 variant lacking exons 3 to 5 in the clone 293FT-75-2 (Figures 9C–E). The effect might be due to the presence of a region within *SNORD75* involved in regulation of splicing of the host gene transcript. Furthermore, the nature of mutations in *SNORD75* in the clone 293FT-75-2 indicates that such regulatory element is located within the chr1:173866903-173866920 region, which corresponds to the deleted sequence in 75-2_9del and 75-2_10del forms. Analysis of *Gas5* splicing events using rMATS (**Supplementary Data Sheet 3**) and JunctionSeq (**Supplementary Data Sheet 2**, **Supplementary Table 2**) revealed numerous changes in the splicing pattern of *Gas5* in 293FT-75-2, while few changes were observed for the other monoclones.

**Figure 10:**
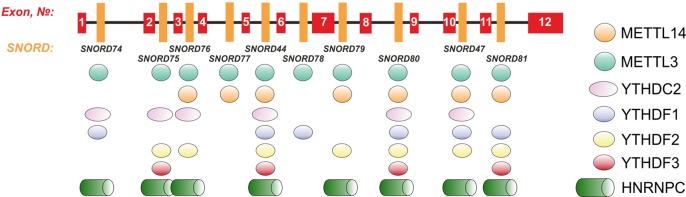
Schematic representation of the binding sites for METTL3/METT14 and m^6^A recognition factors in the *Gas5* gene.

**Figure 11:**
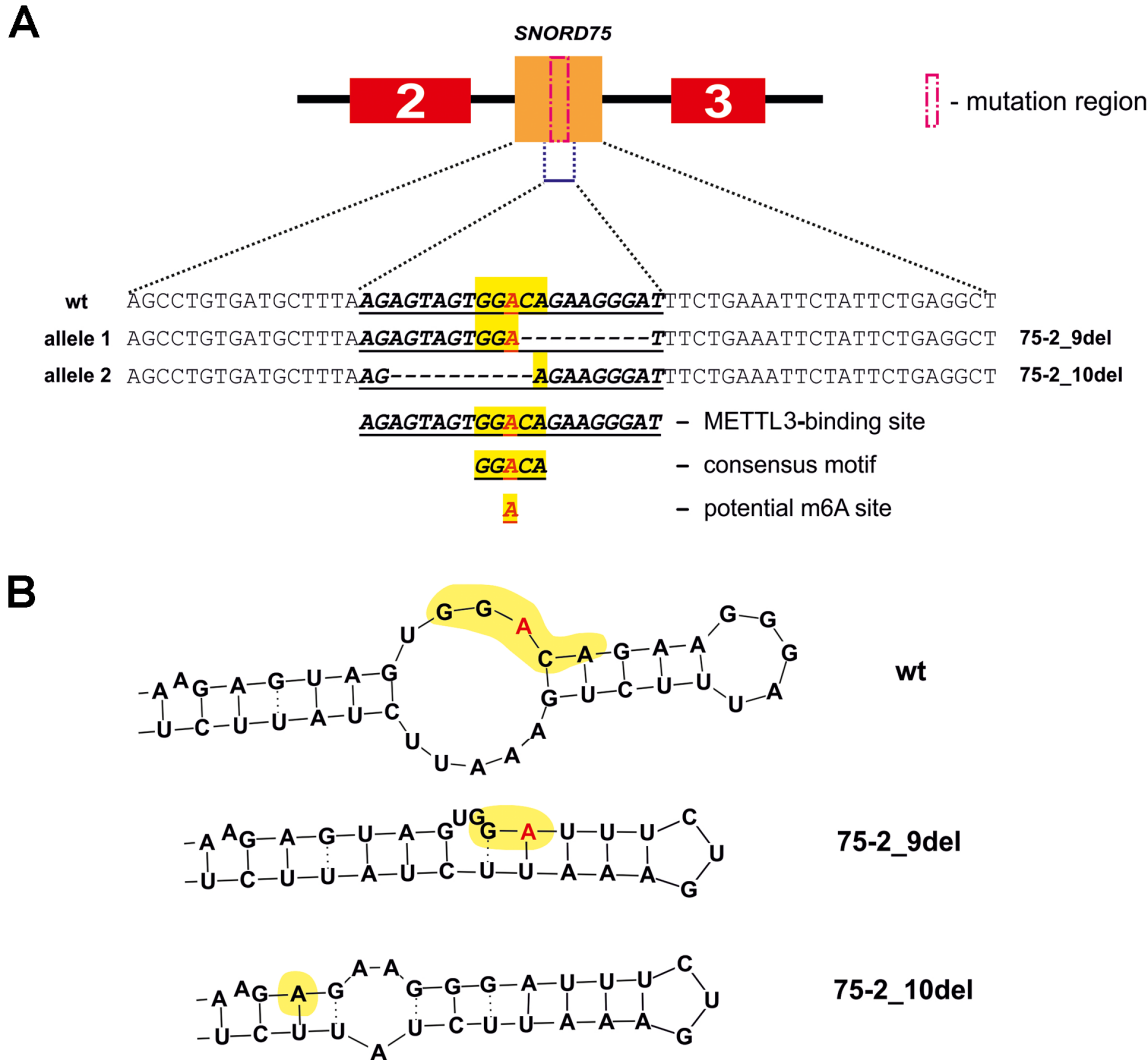
METTL3 binding site in *SNORD75* according to the POSTAR2 database **(A)** and potential secondary stem-loop structures recognized by METTL3 **(B)**. METTL3 binding site in the wild-type and mutant *SNORD75* alleles is shown (bold underline italics). The consensus motif DRACH is highlighted in yellow. The potential N6-methylation site is denoted by red “A” letter.

Further, we analyzed the data on RNA-binding factors interacting precisely with the above-mentioned region within Gas5 lncRNA precursor transcript according to the POSTAR database (Zhu et al., 2019). The following factors were found to be associated with the region in *SNORD75*: METTL3, YTHDF2, YTHDС2, CPSF4, CSTF2T, ELAVL1, FIP1L1, FMR1, HNRNPC, and SSB. Annotation of these proteins revealed that almost all of them were splicing regulatory factors. Therefore, an excision of the regions within Gas5 pre-lncRNA binding some of these proteins might result in formation of the detected products of alternative splicing (**Figure 9**). The presence of binding sites for METTL3/METT14 and a group of m^6^A recognition factors in this region (**Figure 10**) suggests that regulation of Gas5 lncRNA processing is m^6^A-dependent, while formation of alternative splicing products in 293FT-75-2 is due to deletion of one of the m^6^A methylation sites. Moreover, analysis of the *SNORD75* structure demonstrated the presence of the required consensus element DRACH (GGACA in *SNORD75*) and the typical stem-loop structure recognized by m^6^A-methylating complexes, which is absent in 293FT-75-2 due to deletion of the METTL3 recognition region in *SNORD75* (**Figure 11**). Furthermore, bioinformatics analysis of the dataset presented in NCBI GEO (GSE56010) (Liu et al., 2015b) confirmed that the maturation pattern for Gas5 lncRNA alters in similar manner upon knockdown of METTL3, METTL14, and HNRNPC (**Supplementary Tables 2** and **5**, **Supplementary Data Sheet 1**). We found the most numerous alterations in exon and junction coverage after HNRNPC knockdown in the region of 3–8 and 11 exons.

## Discussion

The presented data indicates that snoRNAs can be edited using CRISPR/Cas9 tools with generation of viable cell lines expressing mutant snoRNAs. Our experiments demonstrated that snoRNA genes can be edited accurately, selectively, and efficiently without affecting other snoRNAs encoded within the introns of the same host gene. Mutations can be targeted at the regions of boxes C, D, C,’ and D’ for generation of monoclonal cell lines expressing mutant snoRNA forms. In addition, the region of the Kink-turn area beyond the conserved elements can be also used as the target in snoRNA cleavage experiments. In our experiments, we achieved almost complete downregulation of the wild-type snoRNA forms and a minimum four-fold decrease in the level of mutant snoRNA forms in the obtained monoclonal cell lines compared to the wild-type snoRNAs.

Interestingly, a decrease, but no abrogation of the target rRNA site modification, was observed for all of the obtained monoclones (except for 293FT-77-1) (**Figure 7**, **Supplementary Table 1** **Figures 2** and **3**). Such data indicate that all of the four *Gas5* snoRNAs have back up partners, which guide modification of the same site in case if their “partners” are downregulated for some reason. For instance, U80 backs up the modification of A1521 28S rRNA in 293FT-77-1 in the absence of a functional U77 form and vice versa. Indeed, analysis of the expression of U80 RNA in 293FT-77-1 demonstrated an almost two-fold increase in its level (**Figure 4B**). However, the overall level of mutant U77 RNA forms is decreased significantly in 293FT-80-1 (**Figure 4B**). The existence of more than one snoRNA that guide methylation of the same position is known for several rRNA sites (Watkins et al., 2002; Kehr et al., 2014). In addition, high-throughput RNA sequencing experiments indicate that numerous sites share complementarity with more than one snoRNA (Gumienny et al., 2016; Jorjani et al., 2016). Of course, most of these targets have been only predicted based on sequence complementarity but not yet verified. It is considered that, in case of changed cell conditions or altered expression of a specific snoRNA, another snoRNA backs up the modification for it (Kehr et al., 2014).

The obtained cell lines encoding snoRNAs with modified structure present a convenient and useful model for the study of metabolic pathways involving the target snoRNA. Thus, the presented cell lines, as well as similarly obtained cells, can be used for the study of the role of individual snoRNAs in the regulation of gene expression in human cells. Numerous studies demonstrate that snoRNAs are also involved in regulation of mRNA expression and alternative mRNA splicing (Falaleeva et al., 2016). In addition, some snoRNAs are processed into short miRNA-like derivatives, which perform fine-tuning of some pathways (Ender et al., 2008; Taft et al., 2009; Brameier et al., 2011; (Martens-Uzunova et al., 2015). Hence, the developed strategy allows one to reveal novel non-canonical RNA targets for small nucleolar RNAs, map functionally significant sites of modification within ribosomal RNAs, and create models for elucidation of ribosome heterogeneity phenomenon (Byrgazov et al., 2013; Krogh et al., 2016; Genuth and Barna, 2018). The absence of significant activation/inactivation of key cellular metabolic pathways indicates that snoRNAs can be cleaved selectively without deterioration of essential cellular processes.

Some snoRNAs are known to be splicing-dependent, while others mature independently of the host-gene transcript splicing (Hirose et al., 2003). Apparently, snoRNA genes can also contain splicing regulatory elements and elements important for binding of various splicing regulatory factors. Our study indicates that *SNORD75* contains such element in the following region chr1: 173866903-173866920. Using the POSTAR database, we have found a series of RNA-binding factors interacting precisely with the above-mentioned region within lncRNA Gas5 precursor transcript. One of the identified factors is N6-adenosine-methyltransferase METTL3/METTL14. Furthermore, there are factors, including YTHDF2, YTHDF3, YTHDС2, and HNRNPC, which are known to bind to an m^6^A-modified RNA only, that are also associated with this region, indicating that the region is subjected to m^6^A modification. Recruitment of m^6^A-recognizing-factors in *Gas5* introns suggests possible m^6^A modification of these regions. In the past decade, it has been established that m^6^A is a dynamic regulator of the processes of maturation, export, and degradation of pre-mRNA and lncRNA precursors (Wang et al., 2014; Liu et al., 2015b; Wang et al., 2015; Cao et al., 2016; Zhu et al., 2018; Coker et al., 2019). In addition, there are evidences indicating that snoRNA function can be also regulated through N6-methylation. Such modification of adenosine residue in the D box region, which forms a *trans* sugar/Hoogsteen base pair with guanine residue of the C box, prevents formation of the proper k-turn structure and further binding of the 15.5kDa protein; as a result, no snoRNPs are formed (Huang et al., 2017). Therefore, it is reasonable to suppose that splicing of Gas5 pre-lncRNA is m^6^A-dependent and regulated by methylation of one of the nucleotides in the chr1:173866903-173866920 region located in *SNORD75*.

Indeed, a decrease in the level of the host transcript, Gas5 lncRNA, has been noted for all of the obtained monoclones (**Figure 7**). We suggest that this is due to abrogated processing of Gas5 transcripts, which is due to mutations at the sites recognized by splicing regulatory factors, since changes in the intronic regions more likely affect maturation than transcription and stability of the lncRNA. It is intriguing that the binding sites for METTL3/METTL14 complex and m^6^A recognition proteins were found within (or at least overlap with) the *Gas5* *SNORD*s and other multi-snoRNA host genes encoding lncRNAs (**Figure 10**) (Liu et al., 2015a; Liu et al., 2018; Zhu et al., 2019).

The N6-methylation of adenosine residues at the sites located within introns by the METTL3/METTL14 complex is also known to impede splicing and result in slowly processed introns and alternative splicing (Louloupi et al., 2018). We believe that control of Gas5 lncRNA maturation is m^6^A-dependent. This hypothesis is in accordance with our results of analyzing public dataset (GSE56010) on *METTL3*, *METTL14,* and HNRNPC knockdown (**Supplementary Table 5**, **Supplementary Data Sheet 1**) (Liu et al., 2015b). Using JunctionSeq (Hartley and Mullikin, 2016), we found numerous alterations in exon and junction coverage after METTL3 and HNRNPC knockdown (**Supplementary Data Sheet 1**). It is important to note that changes in the representation of *Gas5* exons and junctions are similar for the cells with knockdown of m6A-recognizing factor HNRNPC and for the cell line carrying mutations in **Supplementary Data Sheet 2** and **3**. Further, analysis of the METTL3-binding sites using POSTAR2 database revealed a binding site (173,866,922…173,866,902) for METTL3 within the deleted region in both mutants (75-2_9del and 75-2_10del) of *SNORD75* in 293FT-75-2 cells. Furthermore, a typical consensus “DRACH”motif (where D stands for G, A, or U; R stands for purine; and H is either U or A) is found in the deleted region of *SNORD75* (GGACA) (**Figure 11**). Thus, we suggest that recruitment of METTL3/METTL14 complex itself in this region plays a crucial role in determining the splicing pattern of Gas5 lncRNA transcript. It is still unknown, whether it is the N6A-methylation or the binding that regulates splicing of *Gas5*. We analyzed all known m^6^A sites in the *Gas5* region, including *Gas5 SNORDs*, presented in MeT-DB V2.0 database, and have not found any methylation sites in *SNORD75*. However, its absence may be due to the fact that the modification changes the stability of this intron in the cells. A peculiar phenomenon was recently observed for another enzyme catalyzing N6A-methylation, METTL16: the dwell-time of the protein at the 3’ UTR region of MAT2A mRNA was shown to have an impact on the target gene splicing (Pendleton et al., 2017). Interestingly, that, according to the authors, it is not the methylation itself that contributes to the splicing of the target MAT2A RNA but the occupancy time of the METTL16 at one of the hairpins in the 3’ UTR of the target transcript (Pendleton et al., 2017). Thus, one can suggest that m6A-modifying factors regulate maturation of pre-mRNA and pre-lncRNA gene by binding to a specific intronic region even without causing N6A-methylation.

In the present study, changes in the sequence of the METTL3/METTL14-binding site resulting in the deletion of the consensus motif in *SNORD75* resulted in formation of an alternative splicing product (**Figure 9A**). Taken together, our data suggests that sites responsible for METTL3/METTL14-dependent regulation of Gas5 lncRNA splicing are located within *SNORDs*.

## Conclusions

Box C/D small nucleolar RNAs can be edited *via* CRISPR/Cas9-mediated cleavage at the regions near the conserved elements boxes C, C,’ D, and D,’ and specific downregulation of a target box C/D snoRNA can be achieved. The 2’-O-methylation level of the target rRNA nucleotide can be modulated through CRISPR/Cas9-mediated knockout of the corresponding snoRNA. Deletion of the terminal region with disruption in the K-turn area even in preservation of the C and D box structures was shown to affect proper snoRNA processing and result in its downregulation. *SNORD75* contains an element for binding of splicing regulatory factors, the deletion of which causes the alterations of Gas5 pre-lncRNA maturation. In the current work, we show that METTL3/METTL14 might be among the factors regulating lncRNA maturation, and that *Gas5* splicing might be m^6^A-dependent due to intronic *SNORDs*.

## Data Availability Statement

The datasets generated for this study can be found in ArrayExpress (www.ebi.ac.uk/arrayexpress), Acession E-MTAB-8269.

## Author Contributions

JF and AM designed and mainly did the study under the supervision of GS and VV. EZ, RM, SM, and TG executed RNA-Seq protocol. DS, EZ, and KA performed analysis of RNA-Seq data. EB and DP provided assistance with RT-PCR experiments. DP performed HPLC-MS/MS analysis. JF, AM, and GS wrote the manuscript. All authors have read and approved the content of the manuscript.

## Funding

The study was supported by the RFBR grant No 18-29-07073 and partially (in method development) by State Budget Program (0245-2019-0001).

## Conflict of Interest

The authors declare that the research was conducted in the absence of any commercial or financial relationships that could be construed as a potential conflict of interest.
