# Supplementary material for: Are Small Nucleolar RNAs “CRISPRable”? A Report on Box C/D Small Nucleolar RNA Editing in Human Cells
Source: Front Pharmacol. 2019 Nov 4;10:1246. doi: 10.3389/fphar.2019.01246 (PMC6856654; doi:10.3389/fphar.2019.01246)

JunctionSeq analysis of the dataset presented in NCBI GEO (GSE56010) (Liu et al., 2015b) confirmed that the maturation pattern for Gas5 lncRNA alters upon knockdown of METTL3, METTL14, and HNRNPC.

Gas5 exons upon knockdown of *HNRNPC*

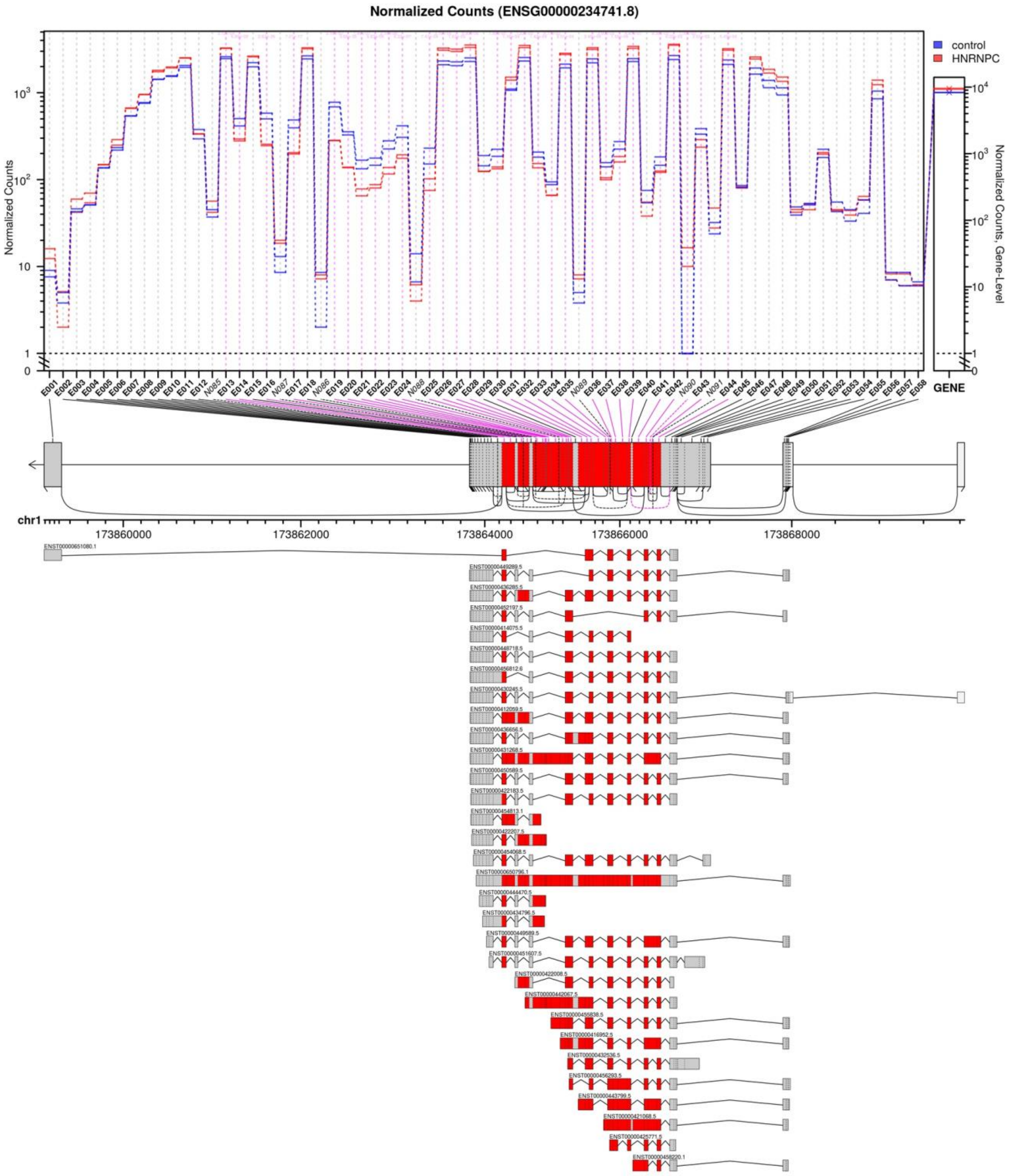

Gas5 junctions upon knockdown of HNRNPC

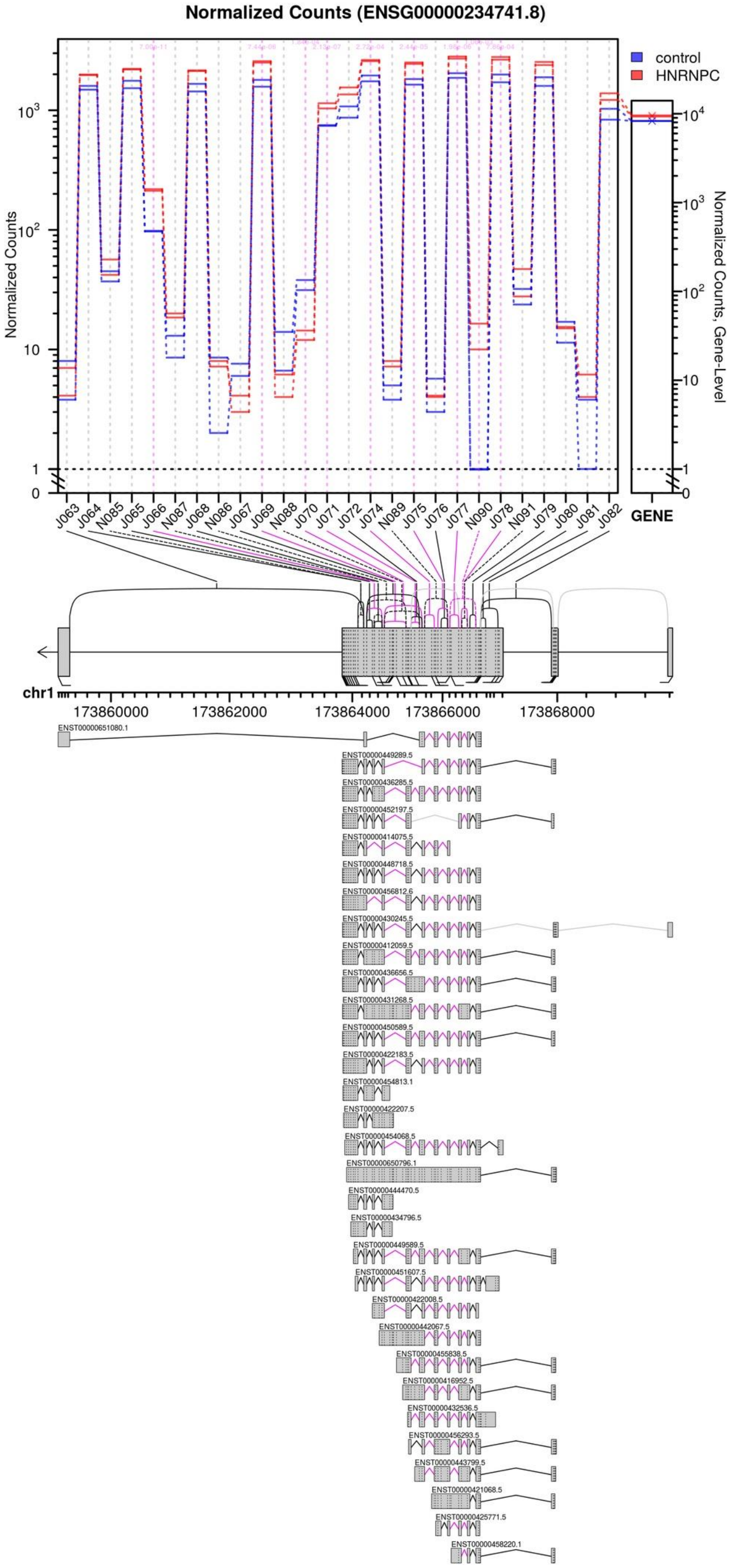

Gas5 exons upon knockdown of *METTL3*

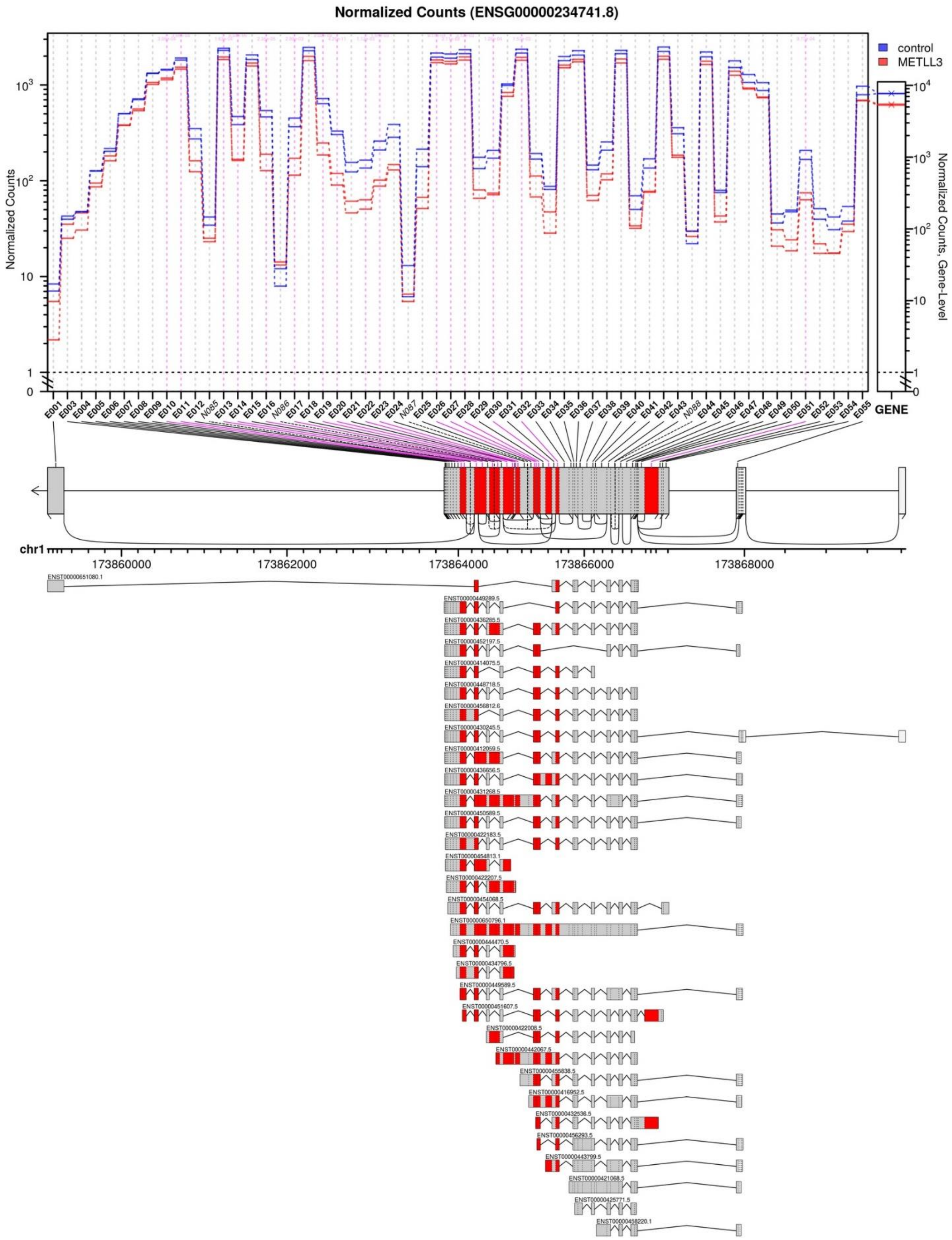

Gas5 junctions upon knockdown of *METTL3*

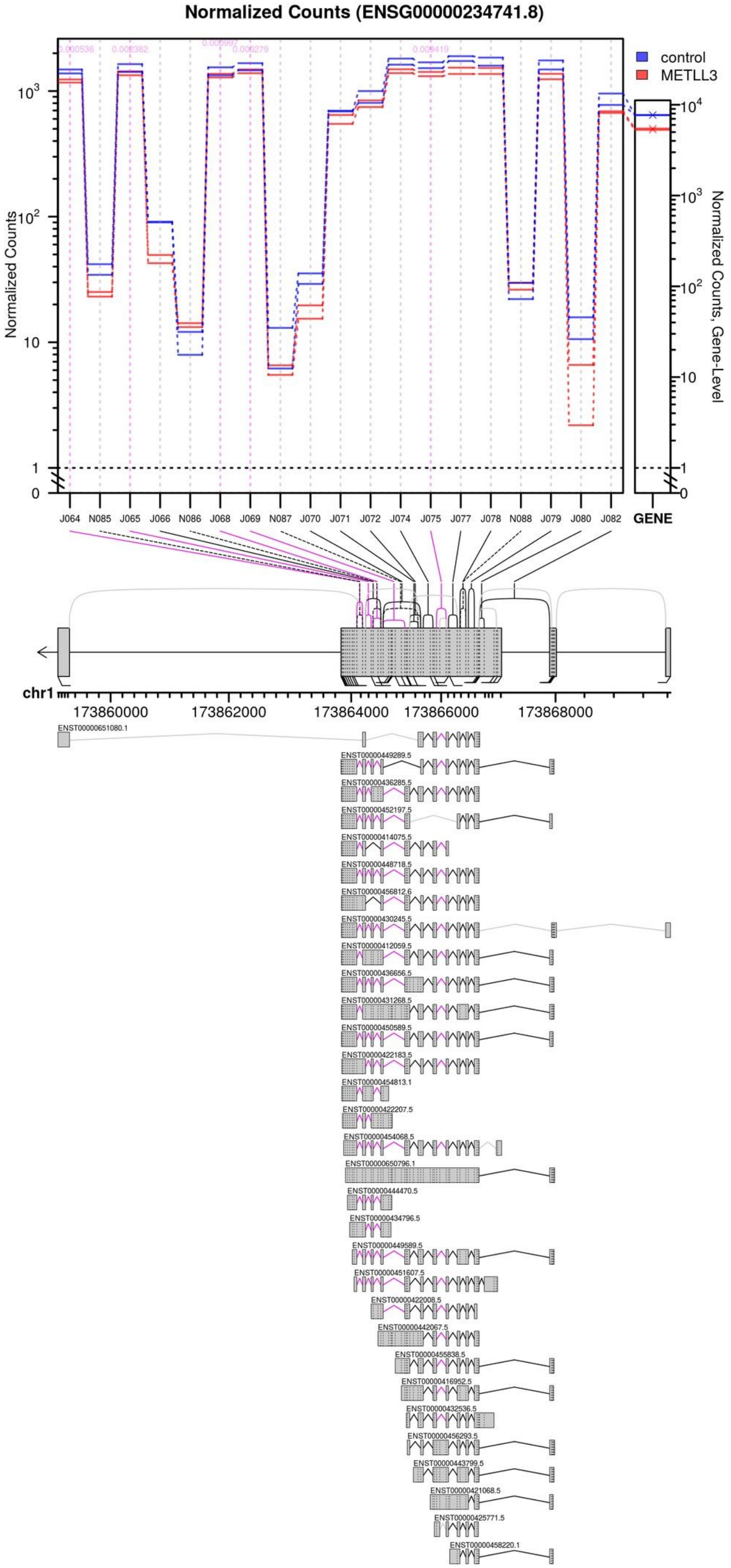

Gas5 exons upon knockdown of *METTL14*

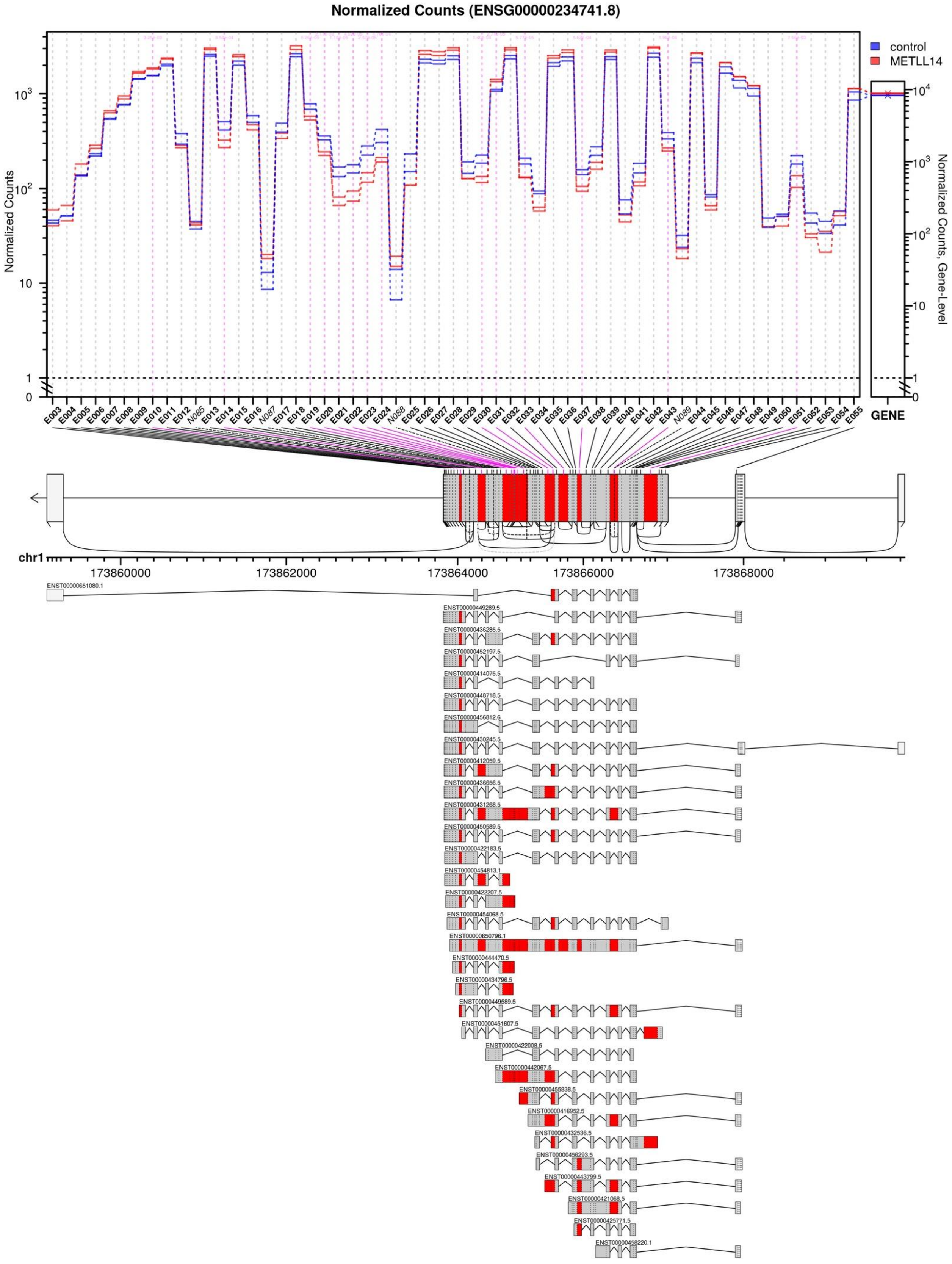

Gas5 junctions upon knockdown of *METTL14*

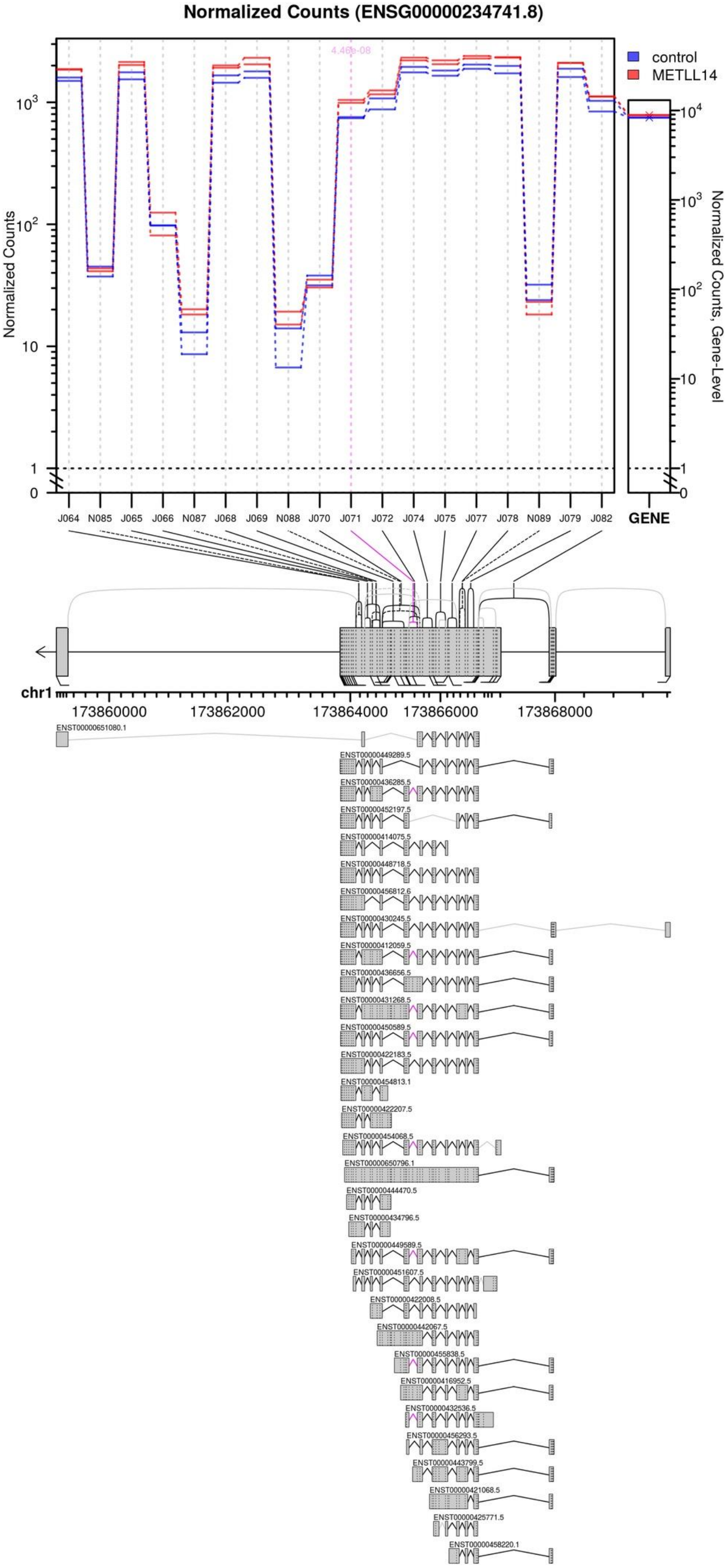

Supplement: Supplementary file 1 [file DataSheet_1.pdf]
