# Supplementary material for: Are Small Nucleolar RNAs “CRISPRable”? A Report on Box C/D Small Nucleolar RNA Editing in Human Cells
Source: Front Pharmacol. 2019 Nov 4;10:1246. doi: 10.3389/fphar.2019.01246 (PMC6856654; doi:10.3389/fphar.2019.01246)

Analysis of *Gas5* splicing events using JunctionSeq revealed numerous changes in the splicing pattern of *Gas5* in 293FT-75-2, while few changes were observed for the other monoclones.

293FT-75-2 vs 293FT-pX

***Gas5* exons**

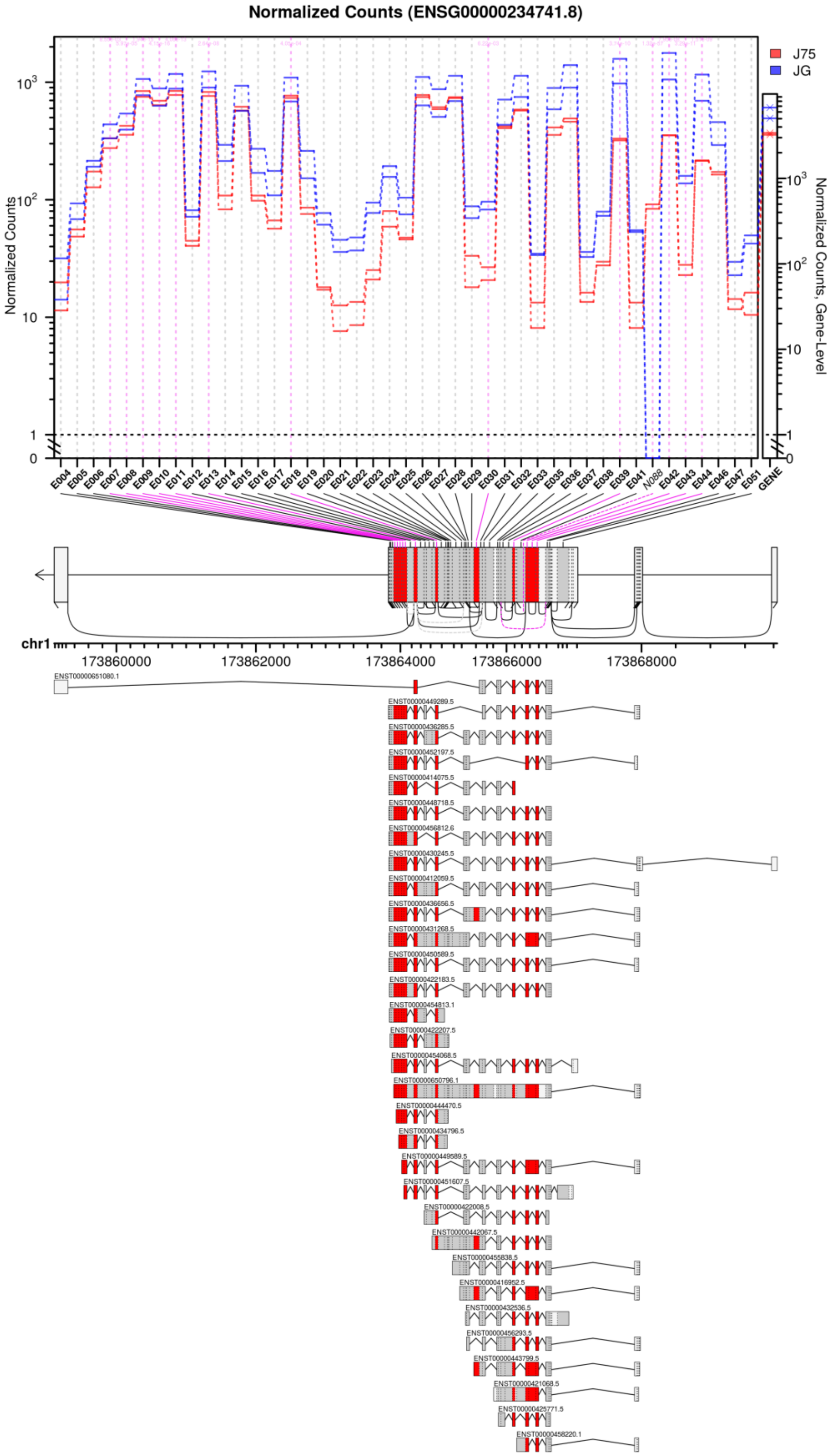

293FT-75-2 vs 293FT-pX

Gas5 junctions

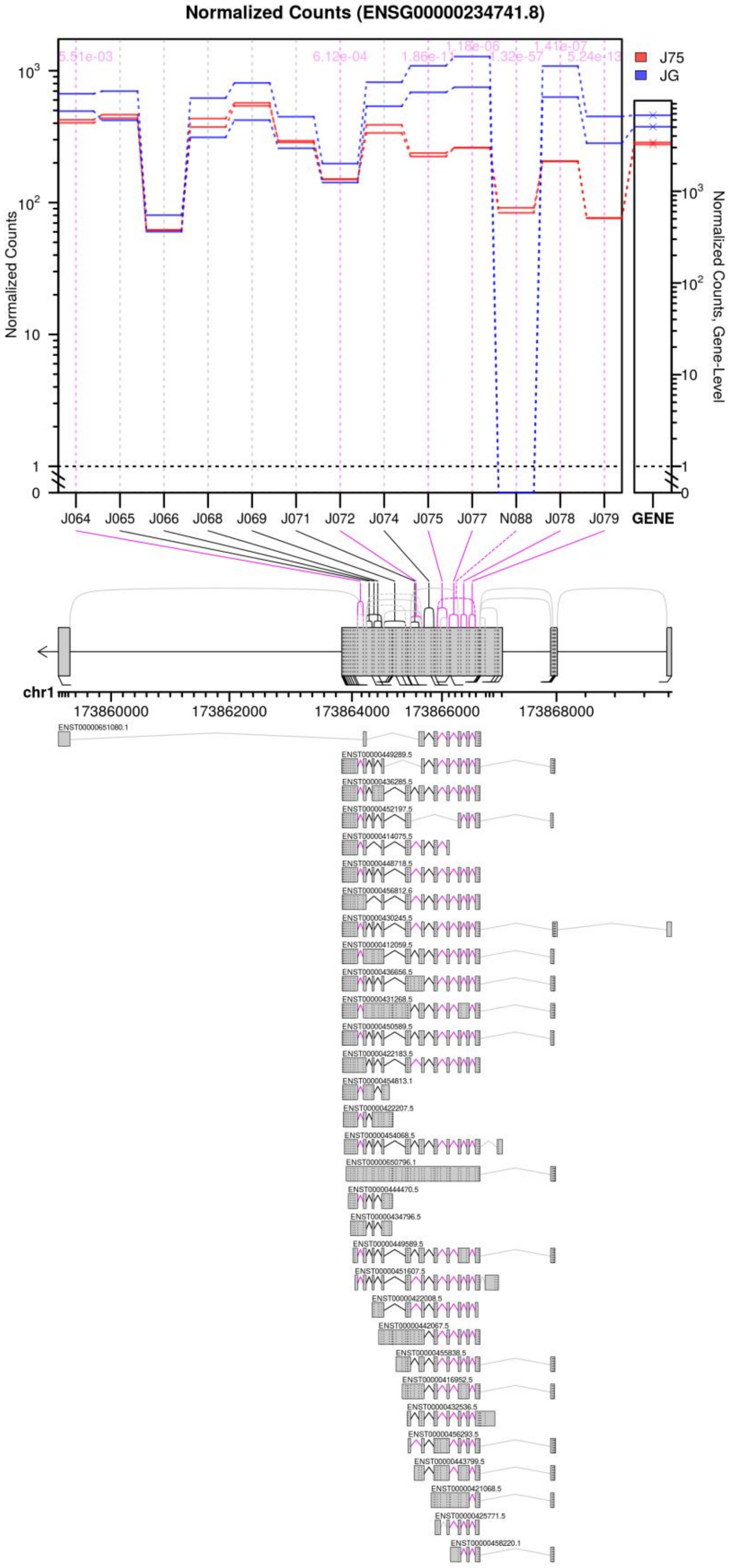

293FT-74-4 vs 293FT-pX

Gas5 exons

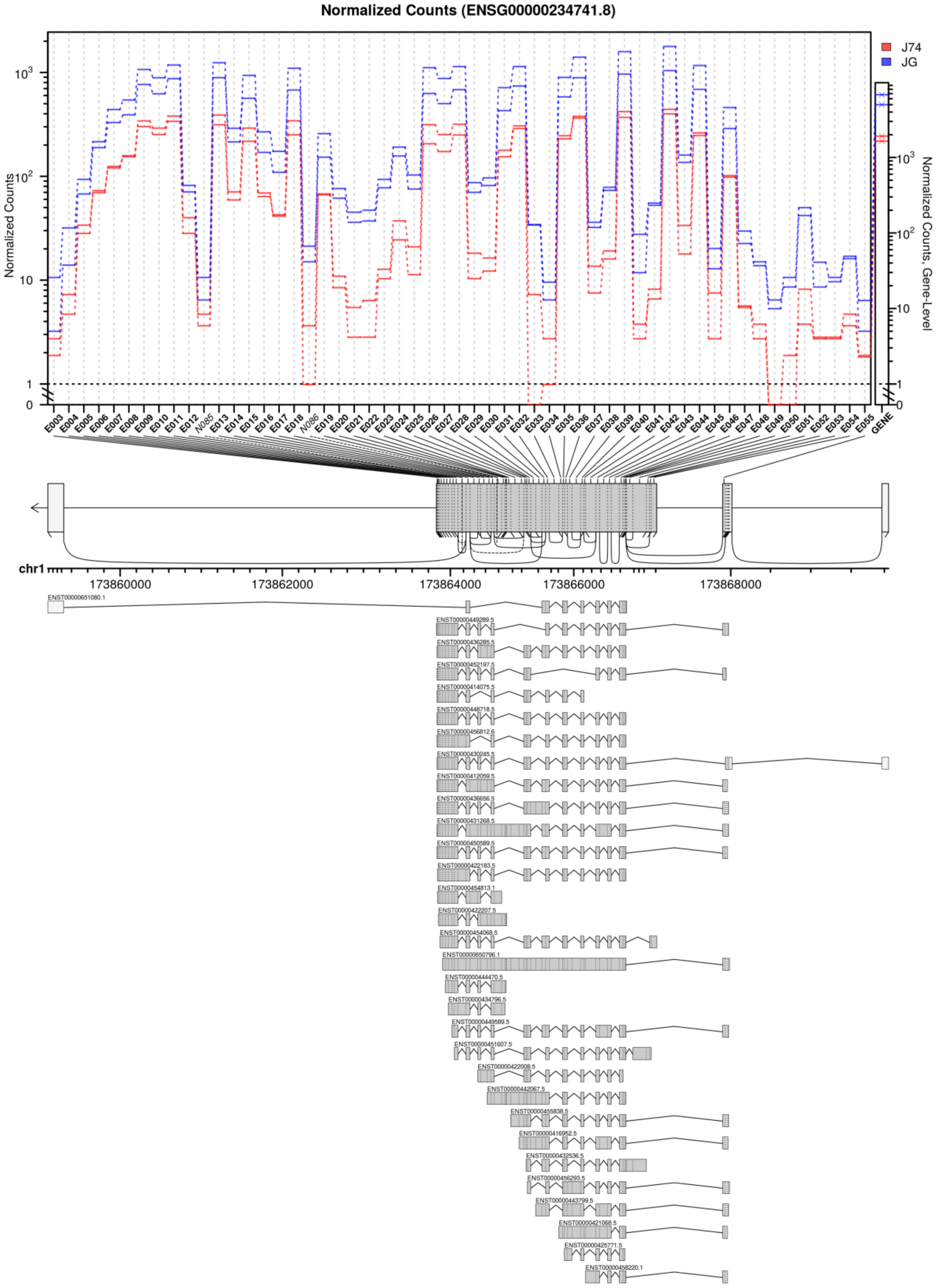

293FT-74-4 vs 293FT-pX

Gas5 junctions

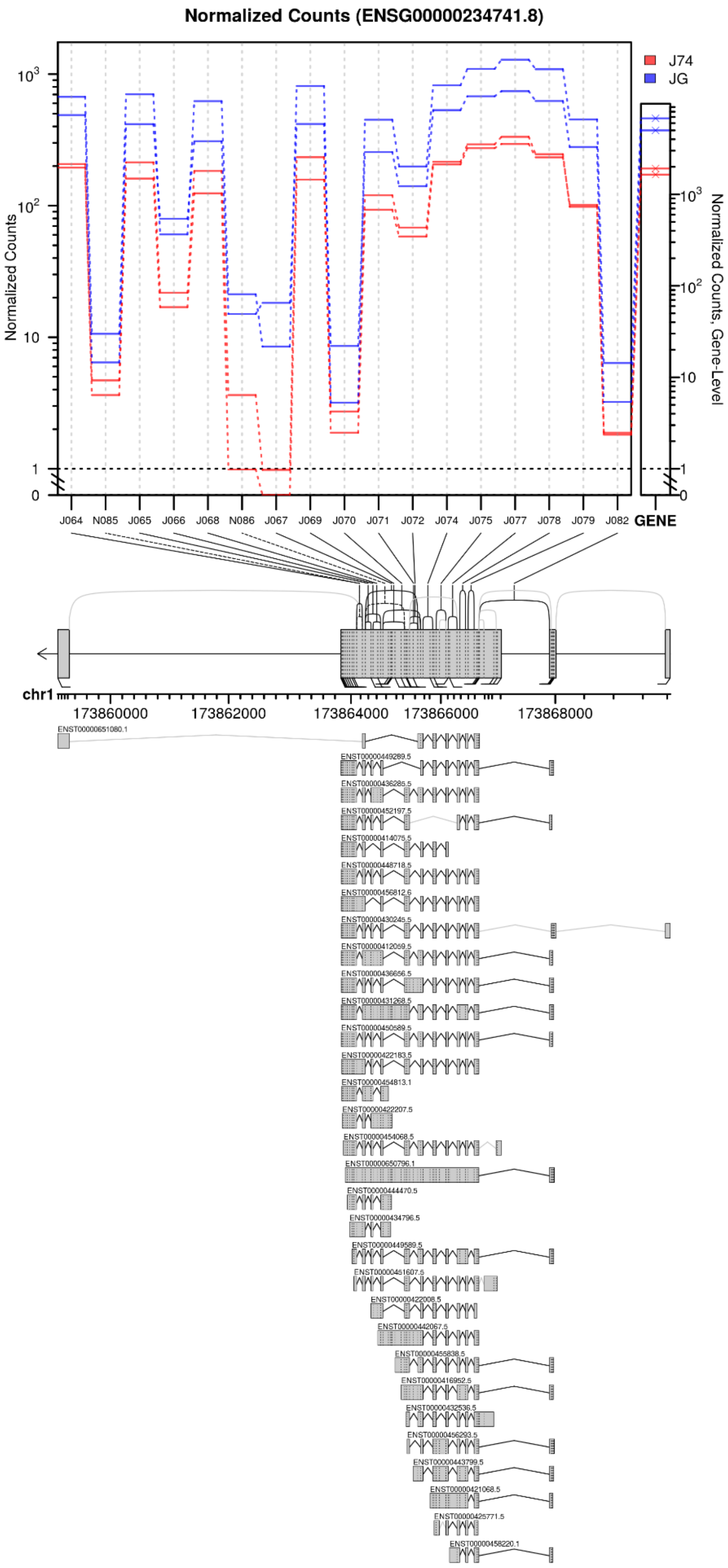

293FT-77-1 vs 293FT-pX

Gas5 exons

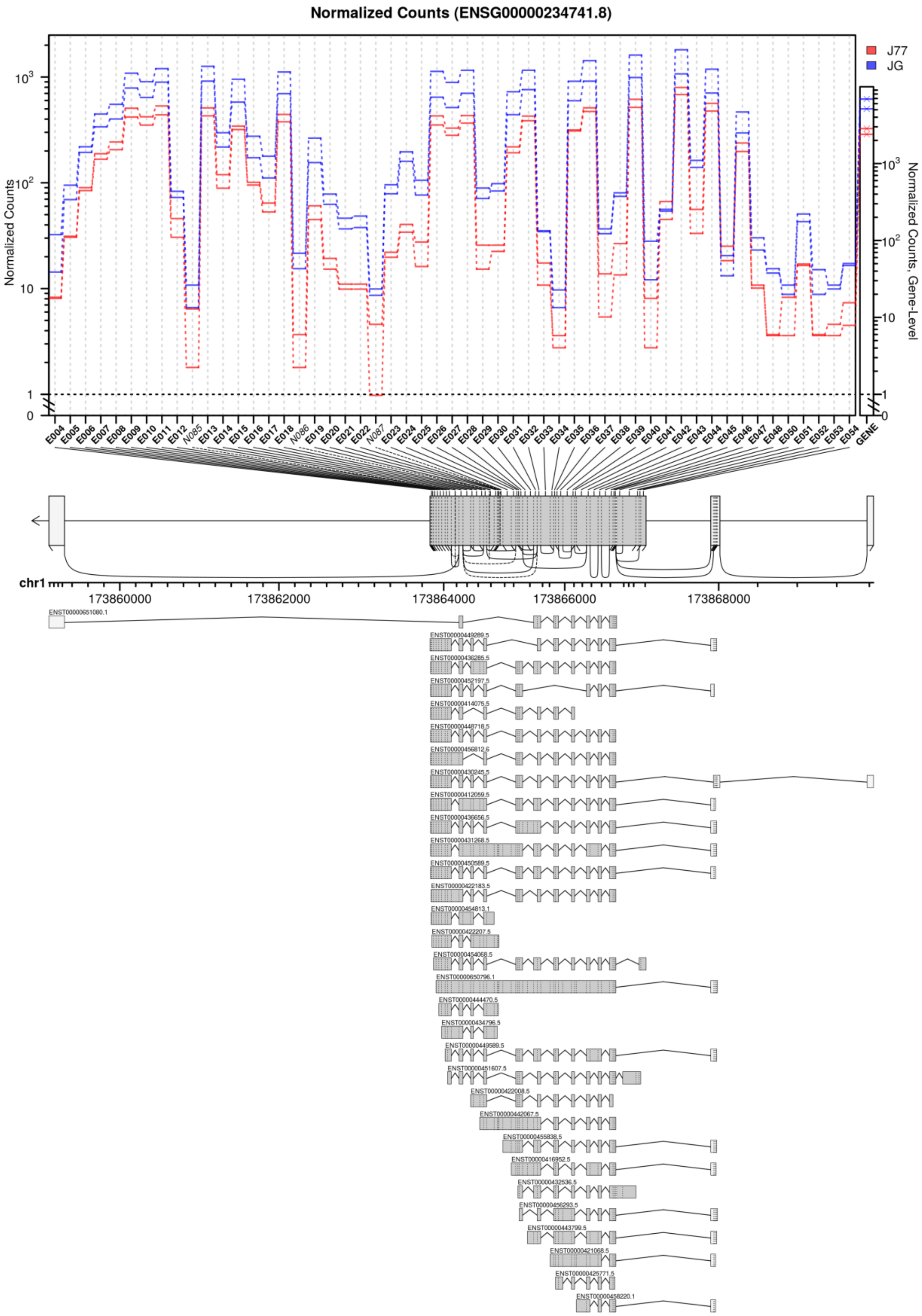

293FT-77-1 vs 293FT-pX

Gas5 junctions

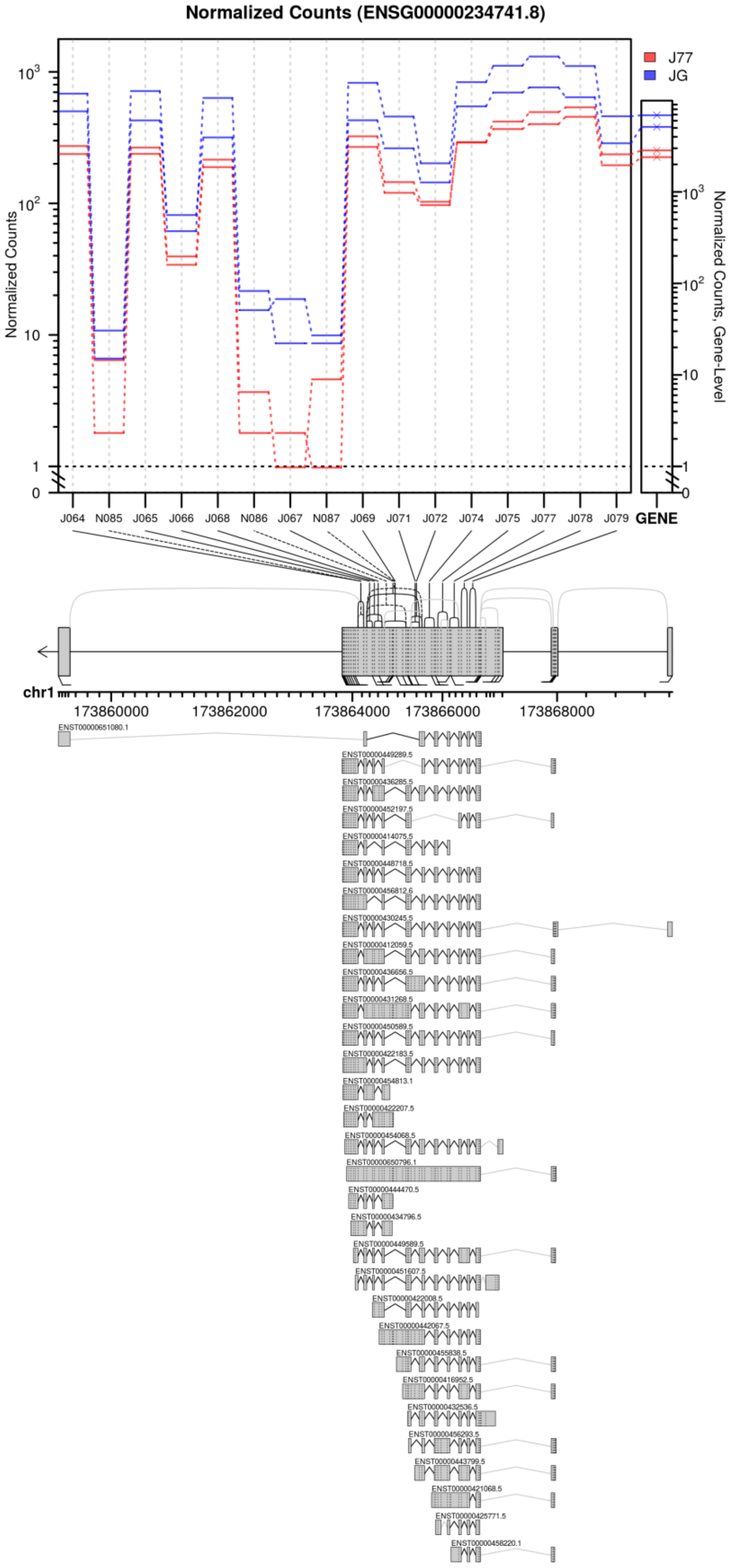

293FT-80-1 vs 293FT-pX

Gas5 exons

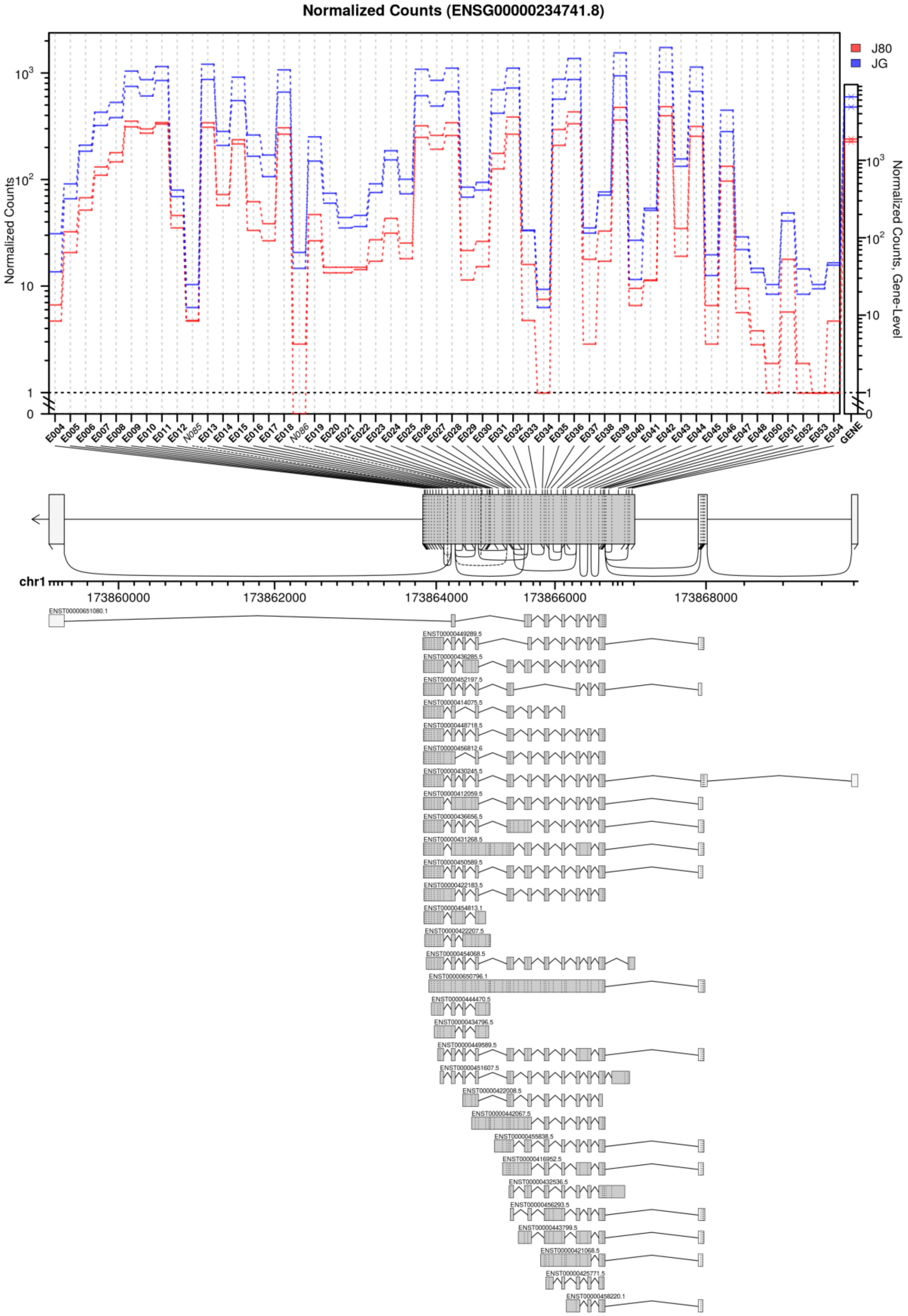

293FT-80-1 vs 293FT-pX

Gas5 junctions

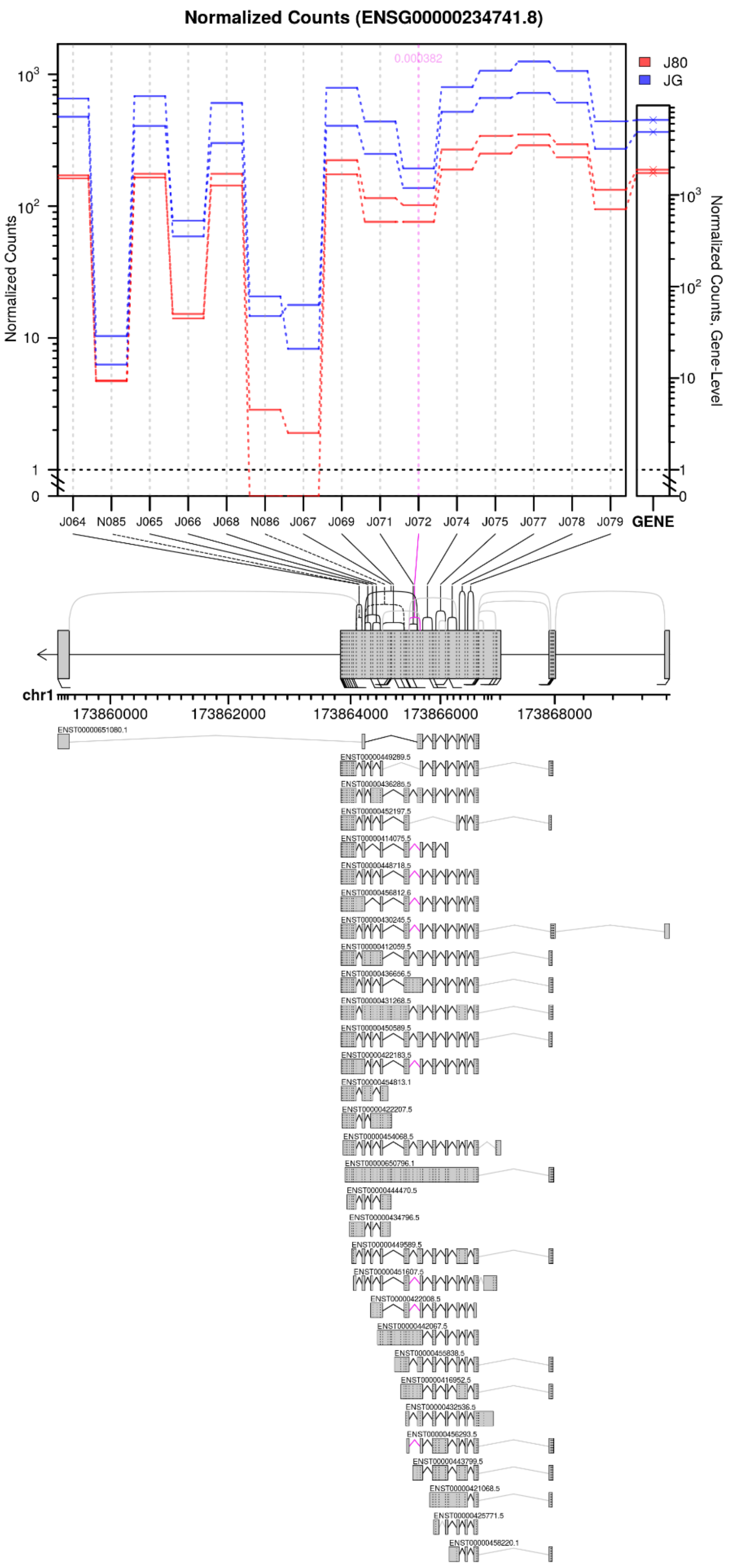

Supplement: Supplementary file 2 [file DataSheet_2.pdf]
