# Supplementary material for: Are Small Nucleolar RNAs “CRISPRable”? A Report on Box C/D Small Nucleolar RNA Editing in Human Cells
Source: Front Pharmacol. 2019 Nov 4;10:1246. doi: 10.3389/fphar.2019.01246 (PMC6856654; doi:10.3389/fphar.2019.01246)
Supplement: Supplementary file 3 [file DataSheet_3.pdf]

Alternative Splicing Events:

SE - Skipped exon

A5SS - Alternative 5' splice site

A3SS - Alternative 3' splice site

MXE - Mutually exclusive exons

RI - Retained intron

Samples:

293FT-pX - 293FT cells transfected with pX458 plasmid without sgRNA

293FT-74-4 - 293FT cells with CRISPR/Cas9 mediated mutation of SNORD74

293FT-75-2 - 293FT cells with CRISPR/Cas9 mediated mutation of SNORD75

293FT-77-1 - 293FT cells with CRISPR/Cas9 mediated mutation of SNORD77

293FT-80-1 - 293FT cells with CRISPR/Cas9 mediated mutation of SNORD80

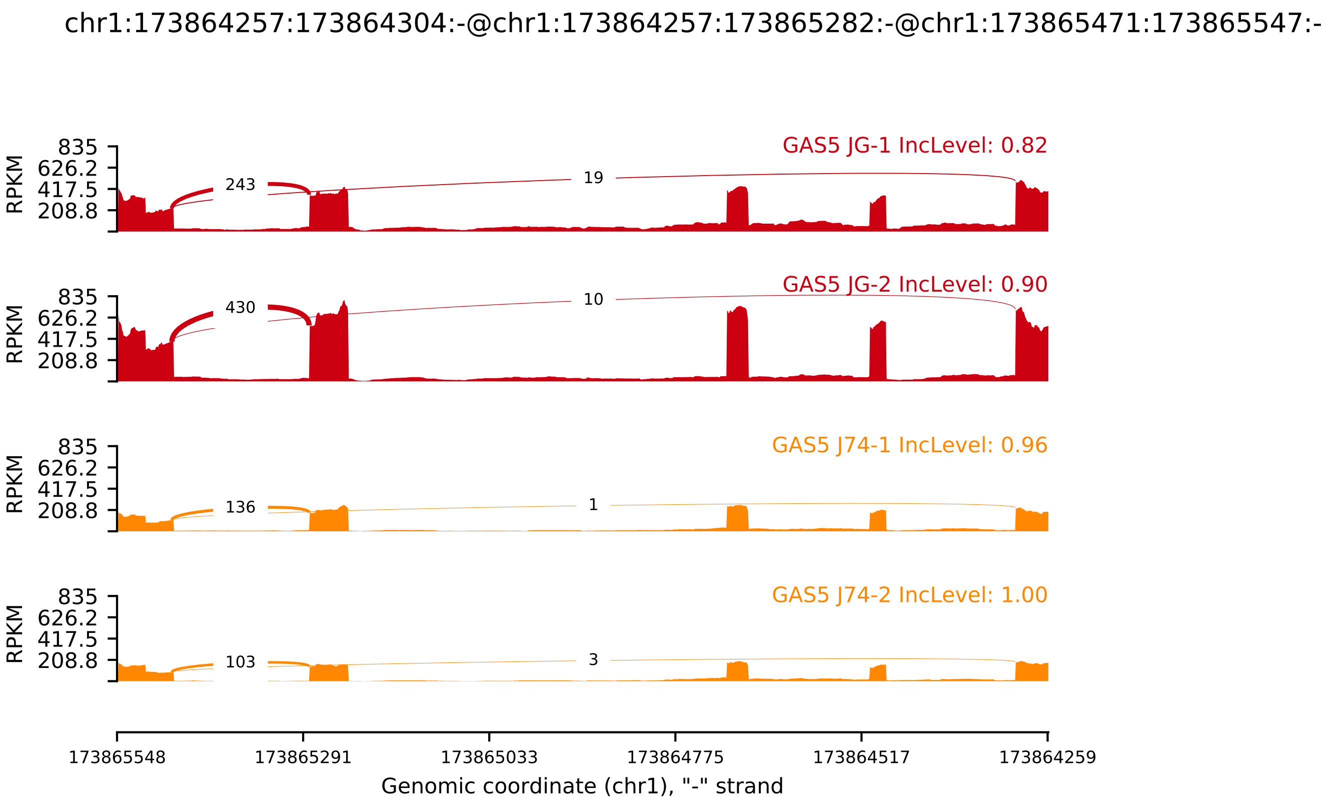

293FT-pX vs 293FT-74-4

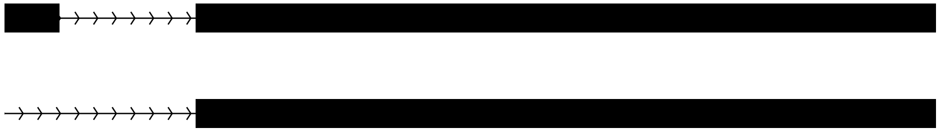

A3SS

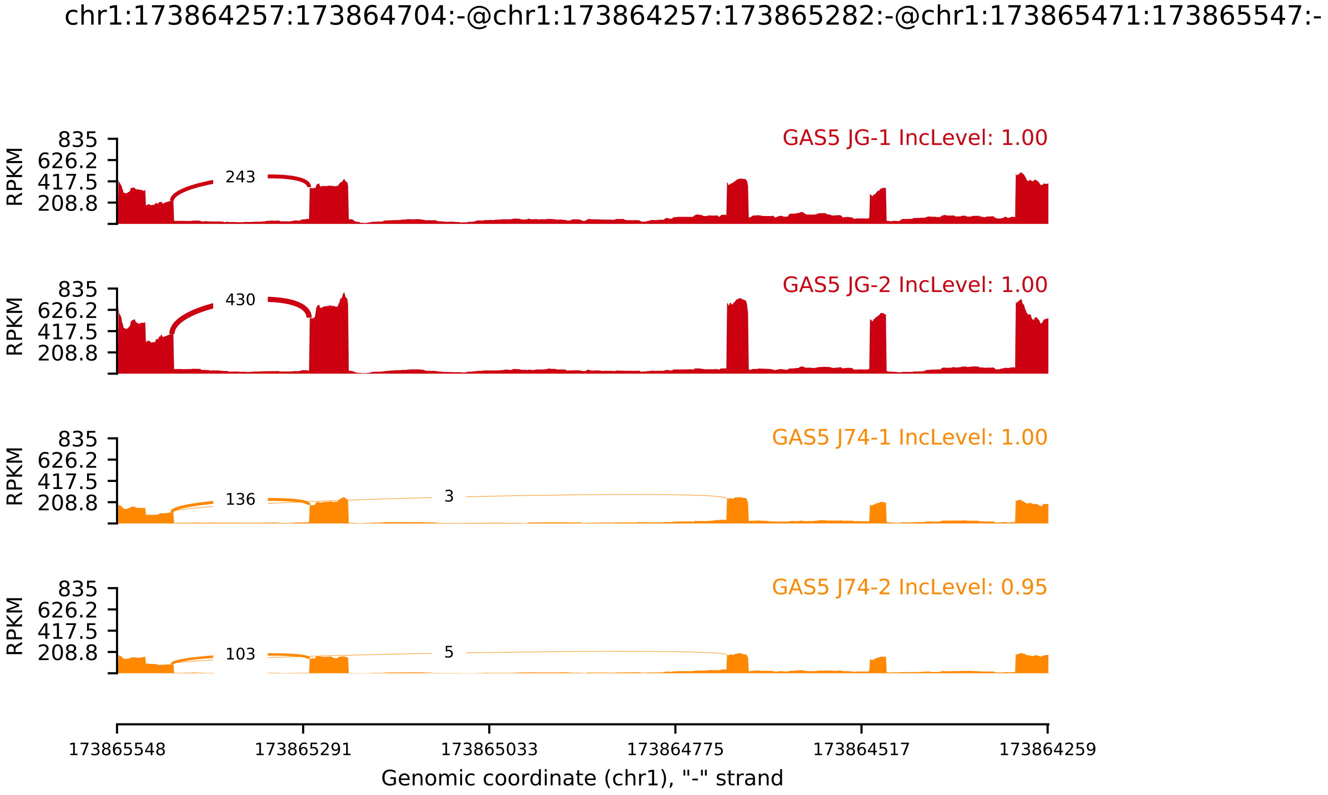

293FT-pX vs 293FT-74-4

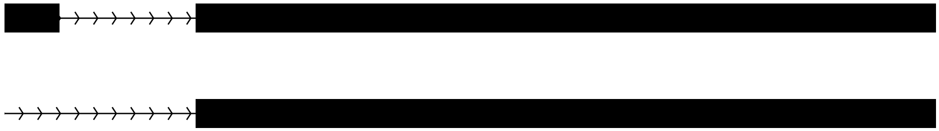

A3SS

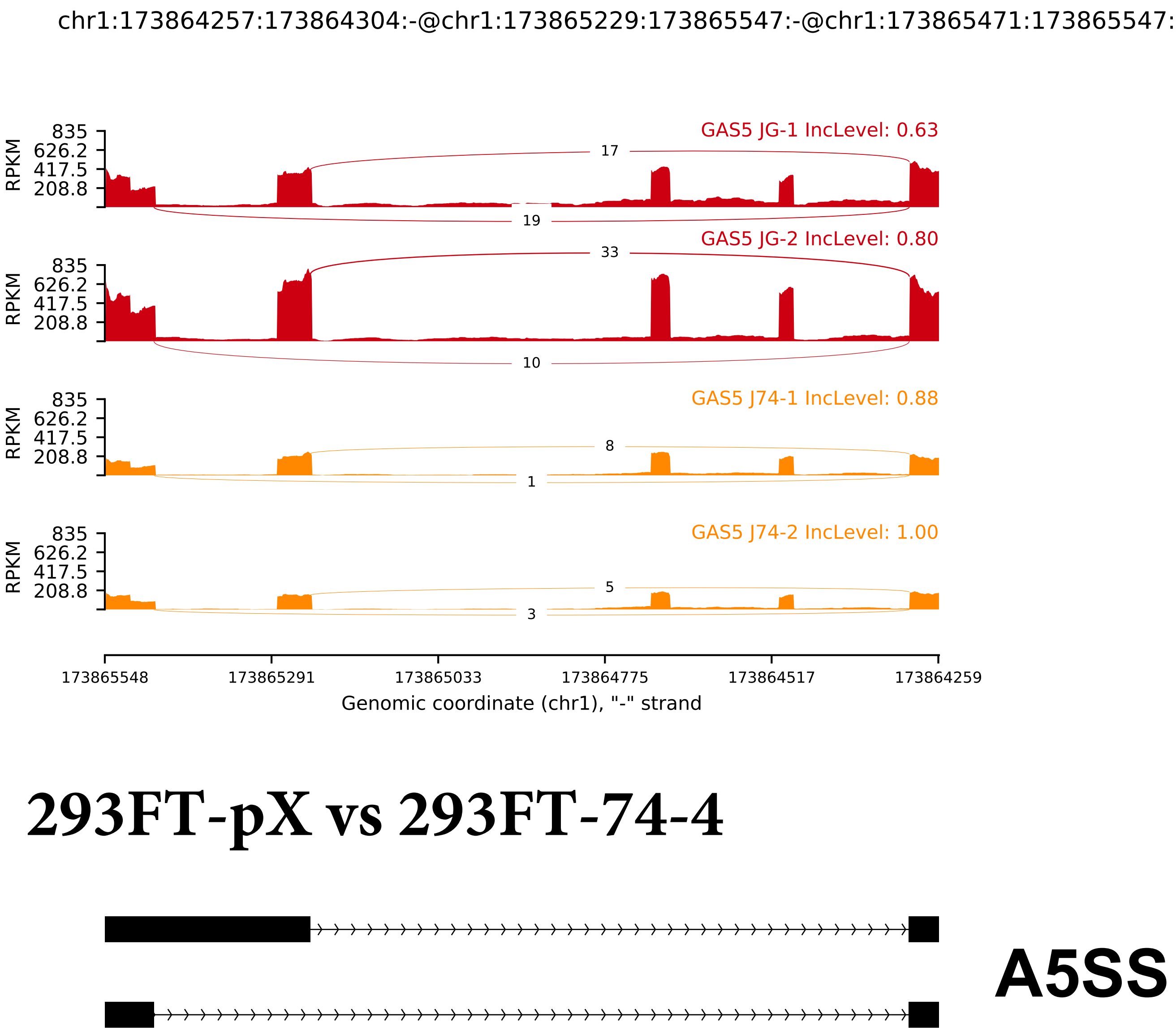

chr1:173864257:173864304:-@chr1:173865229:173865547:-@chr1:173865471:173865547:-
GAS5 JG-1 IncLevel: 0.63
GAS5 JG-2 IncLevel: 0.80
GAS5 J74-1 IncLevel: 0.88
GAS5 J74-2 IncLevel: 1.00
RPKM
835
626.2
417.5
208.8
17
19
33
10
8
1
5
3
173865548
173865291
173865033
173864775
173864517
173864259
Genomic coordinate (chr1), "-" strand
293FT-pX vs 293FT-74-4
A5SS

chr1:173865229:173865282:-@chr1:173865471:173865547:-@chr1:173865510:173865547:-

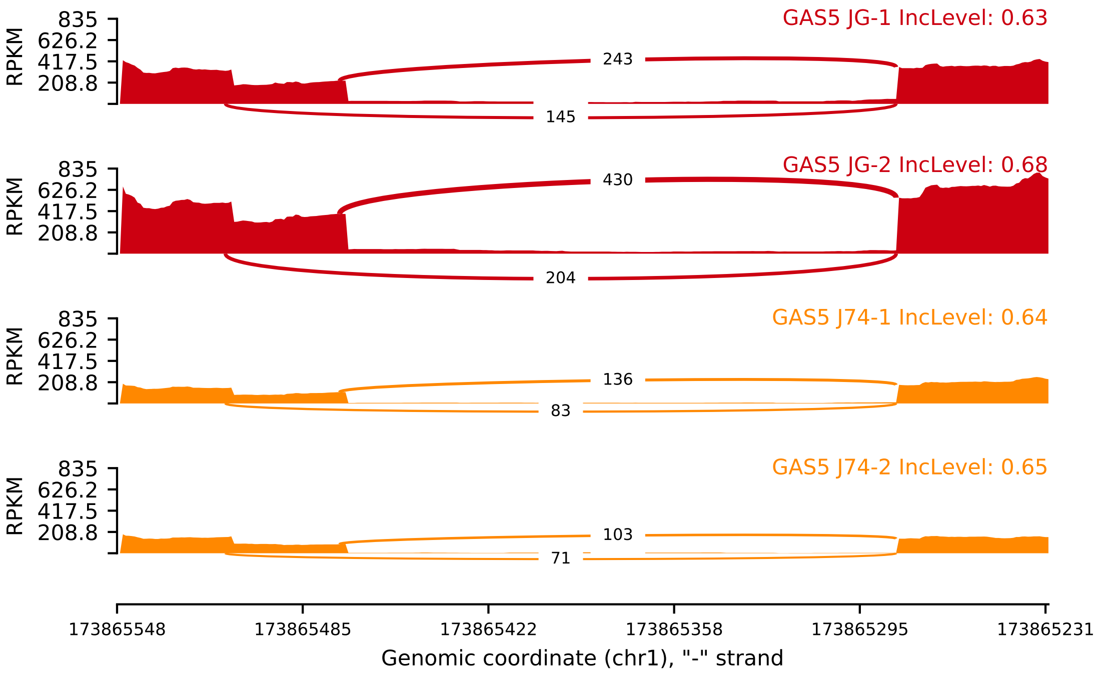

293FT-pX vs 293FT-74-4

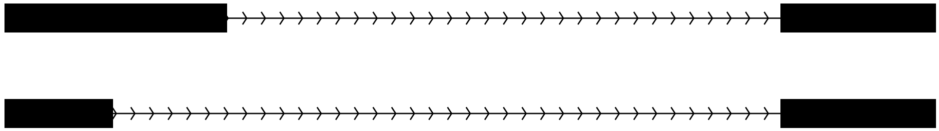

A5SS

chr1:173865471:173865547:-@chr1:173864484:173864704:-@chr1:173864257:173864304:-

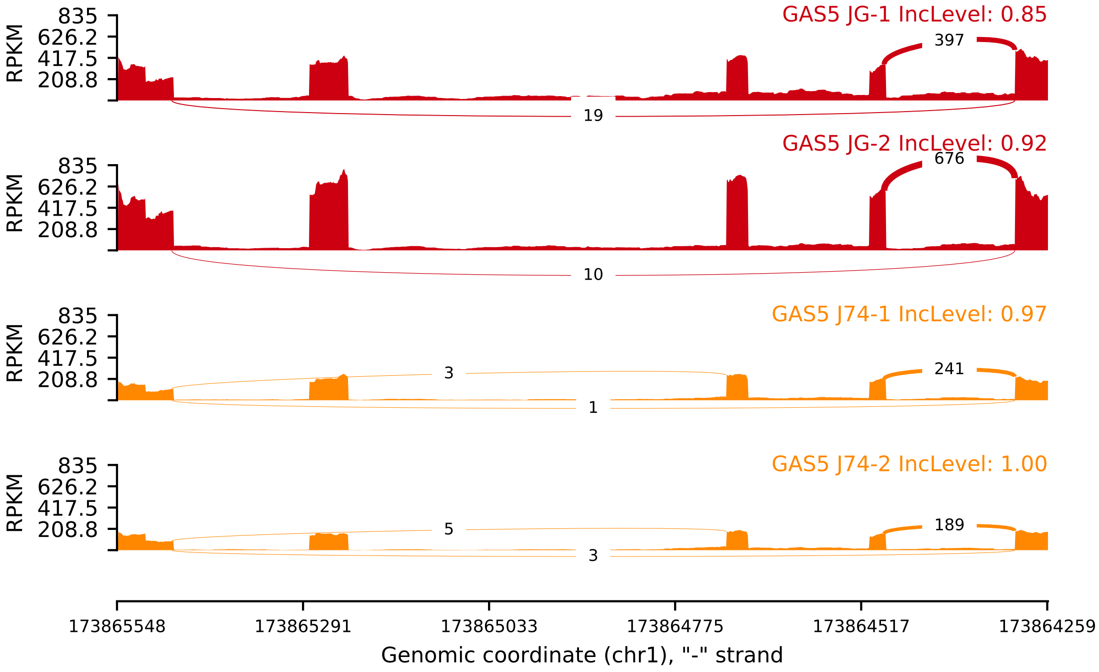

293FT-pX vs 293FT-74-4

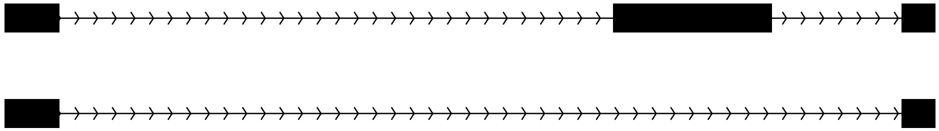

SE

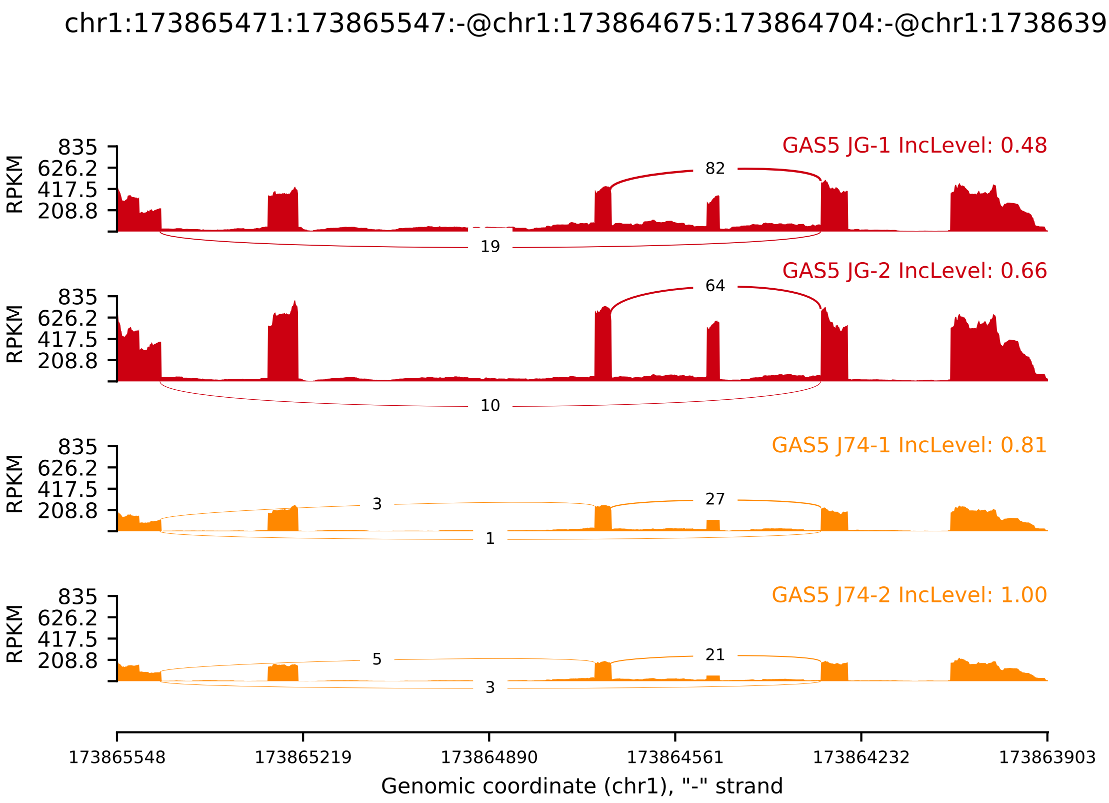

293FT-pX vs 293FT-74-4

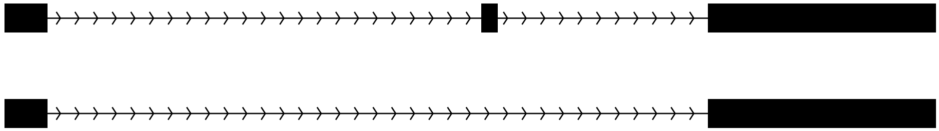

SE

173866991:173867030:-@chr1:173866761:173866796:-@chr1:173865510:173865547:-@chr1:173865229:173865282:-

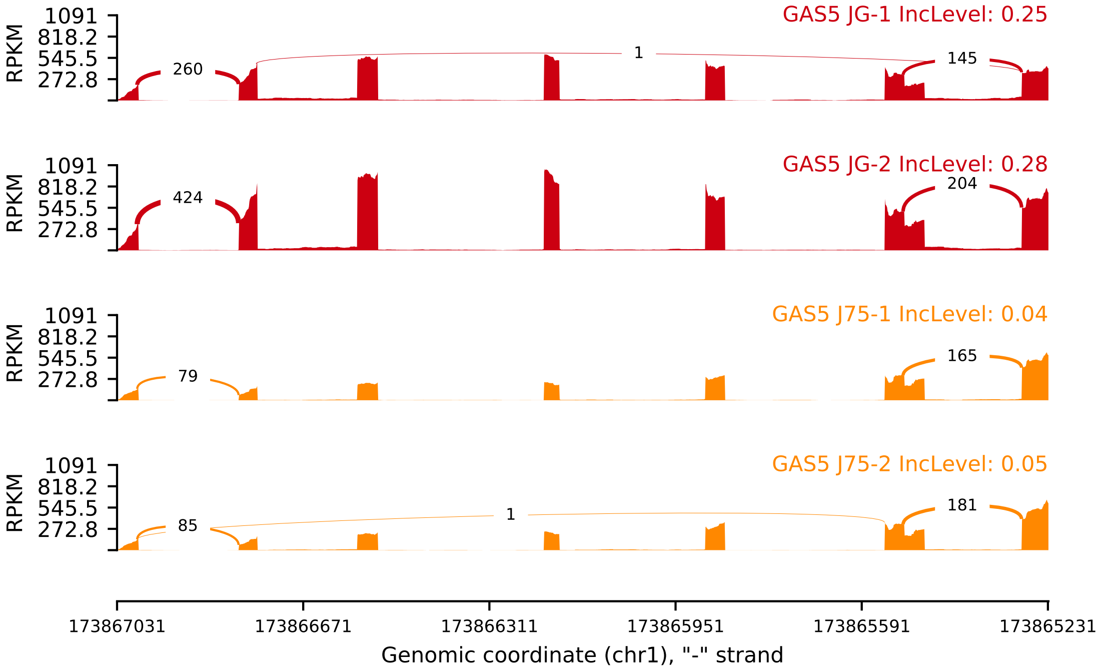

293FT-pX vs 293FT-75-2

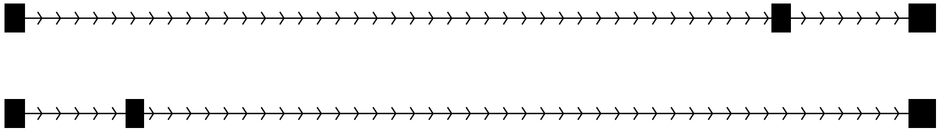

MXE

chr1:173864675:173864704:-@chr1:173864257:173864704:-@chr1:173864257:173864304:-

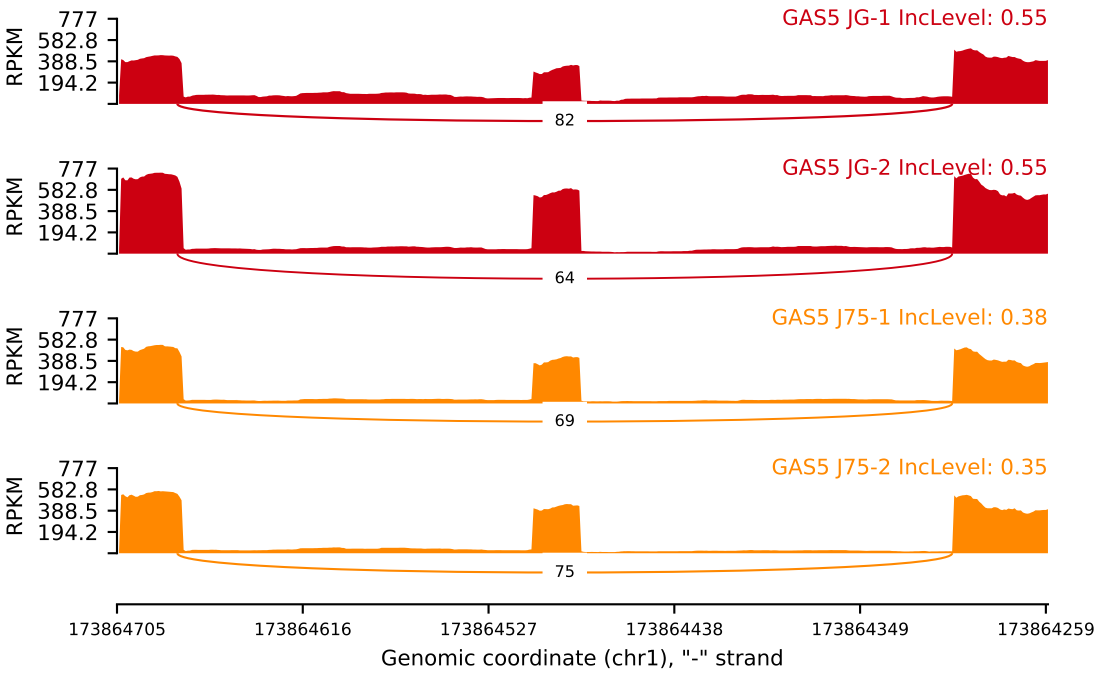

293FT-pX vs 293FT-75-2

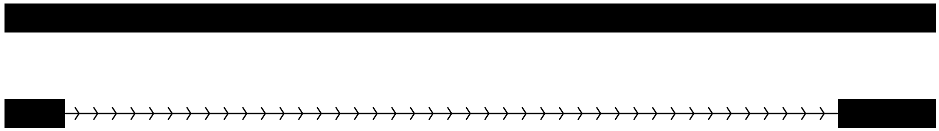

RI

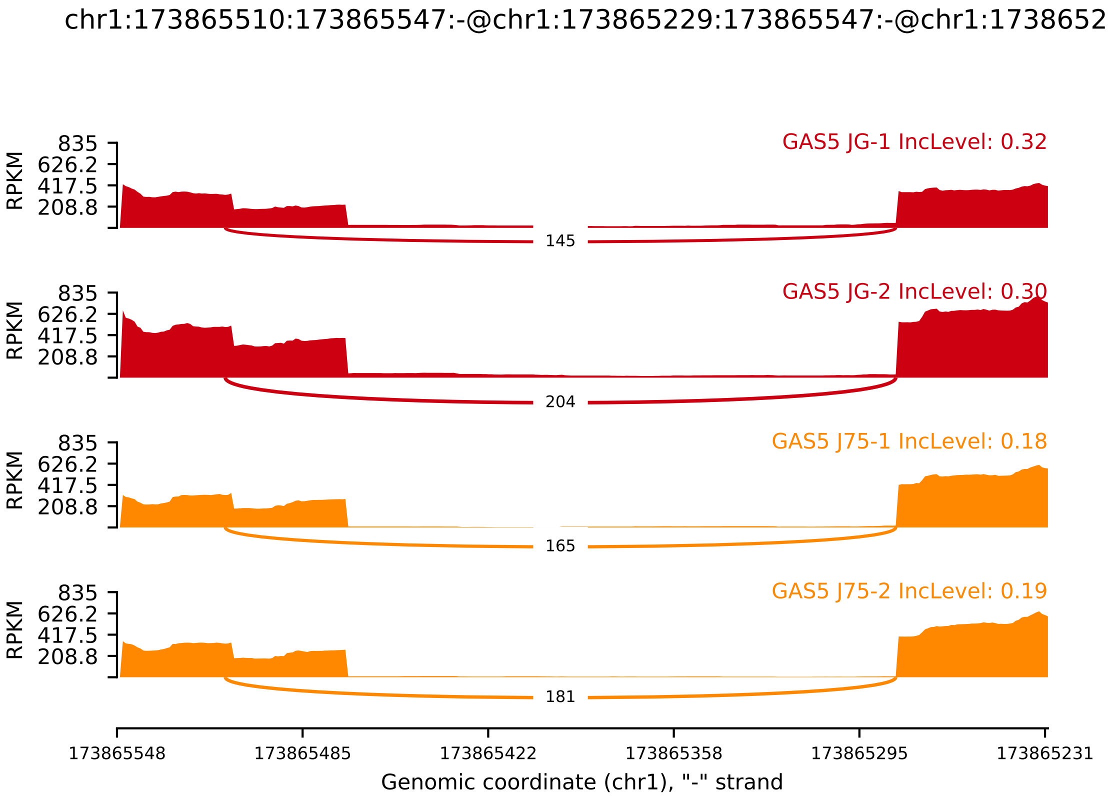

293FT-pX vs 293FT-75-2

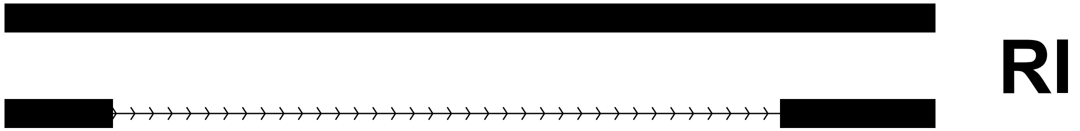

chr1:173866991:173867043:-@chr1:173866528:173866796:-@chr1:173865857:173865894:-

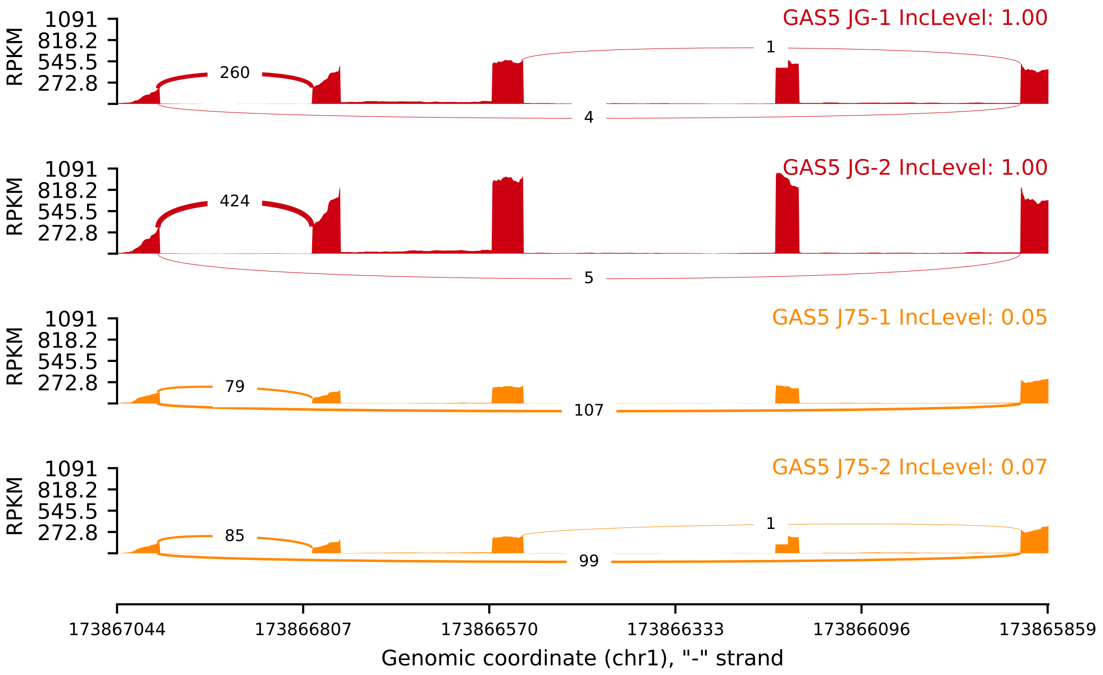

293FT-pX vs 293FT-75-2

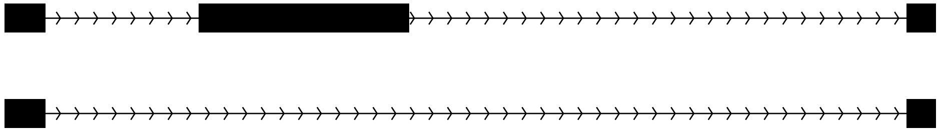

SE

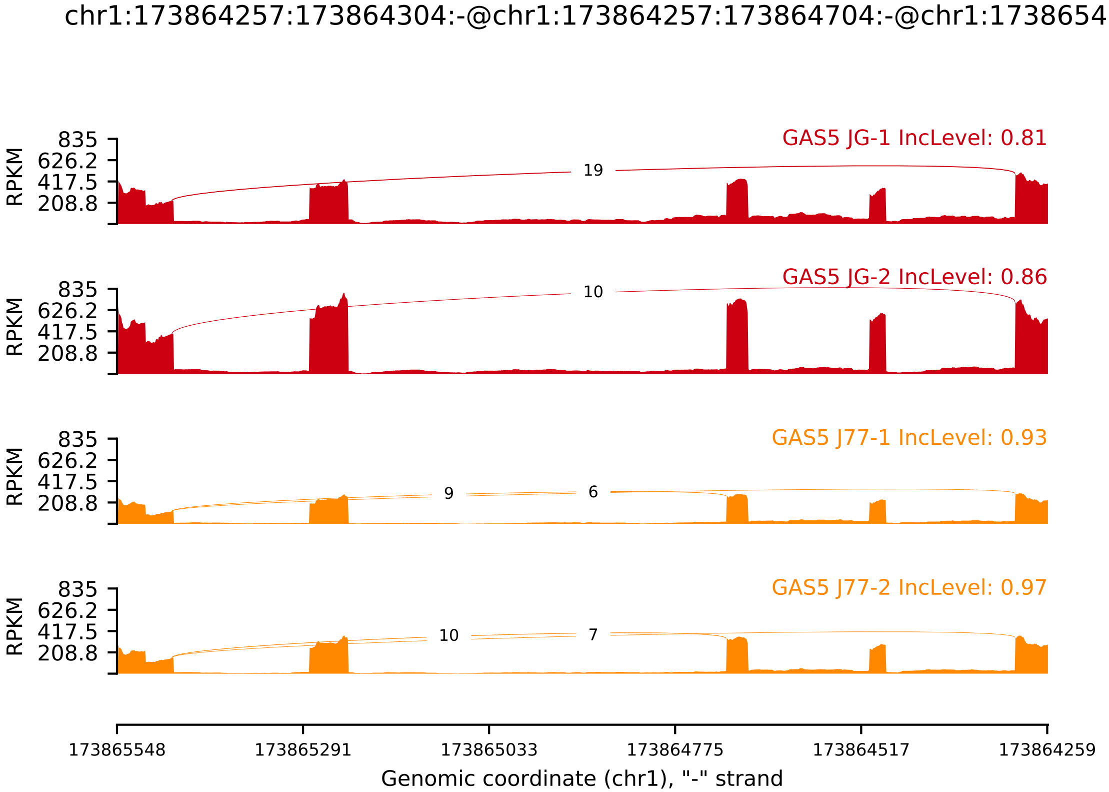

293FT-pX vs 293FT-77-1

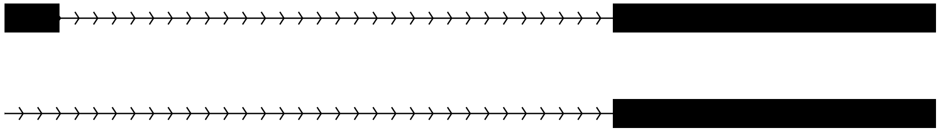

A3SS

chr1:173865510:173865547:-@chr1:173865229:173865547:-@chr1:173865229:173865282:-

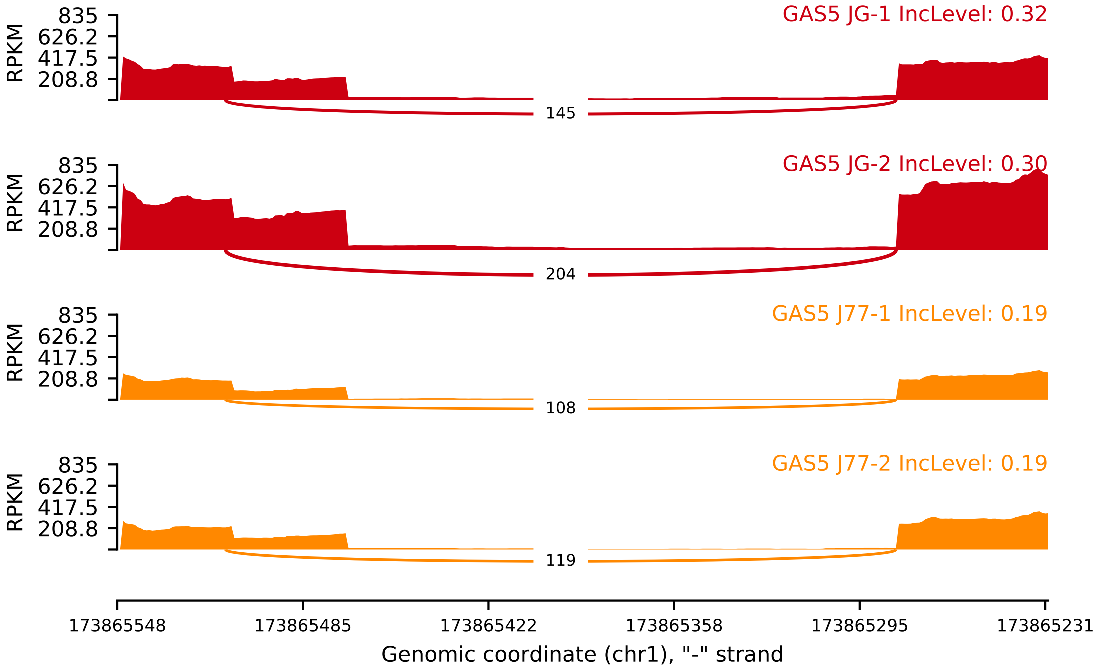

293FT-pX vs 293FT-77-1

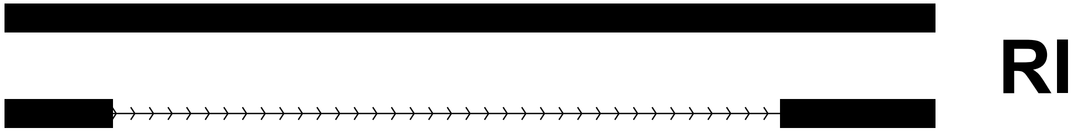

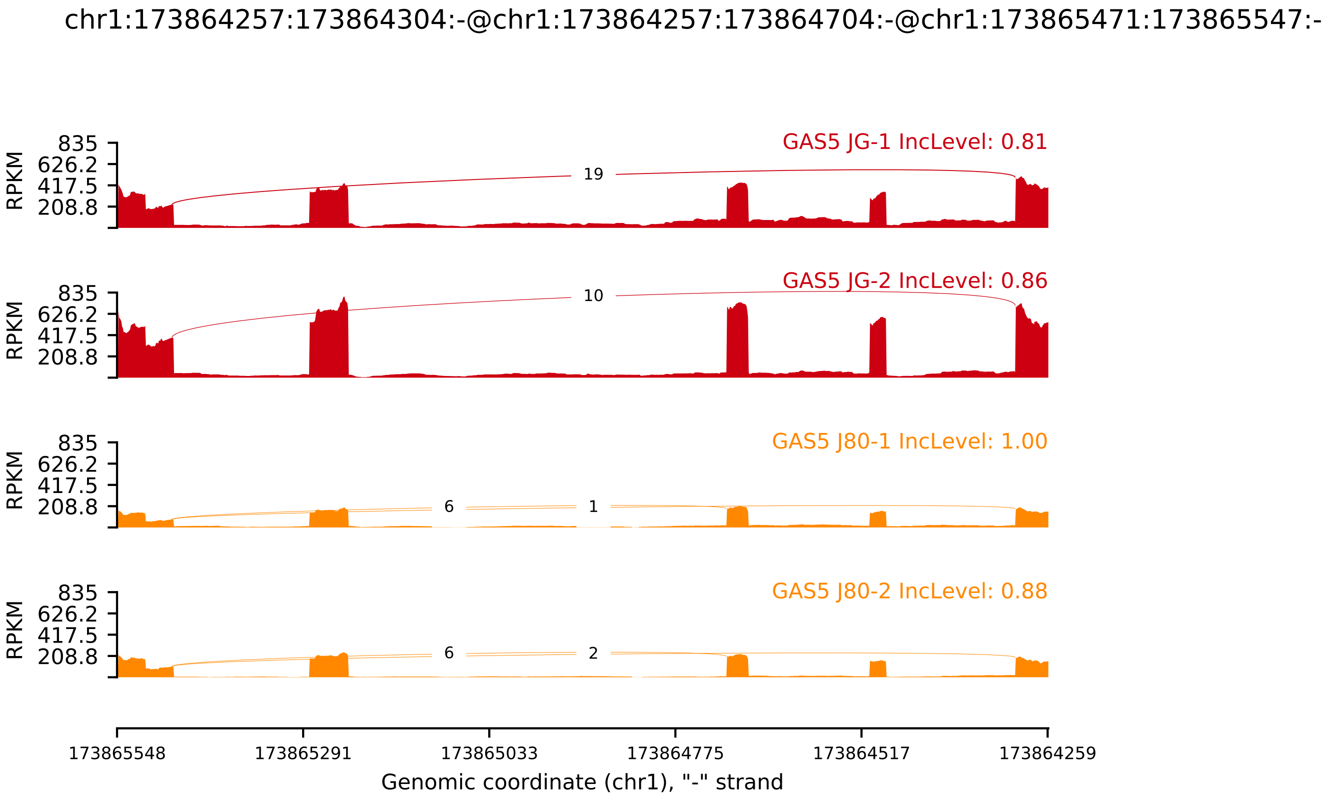

293FT-pX vs 293FT-80-1

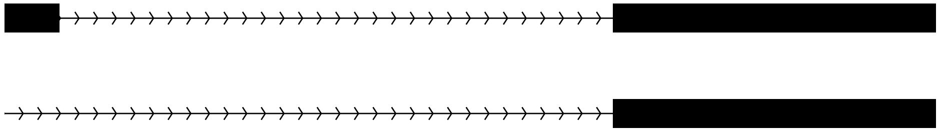

A3SS

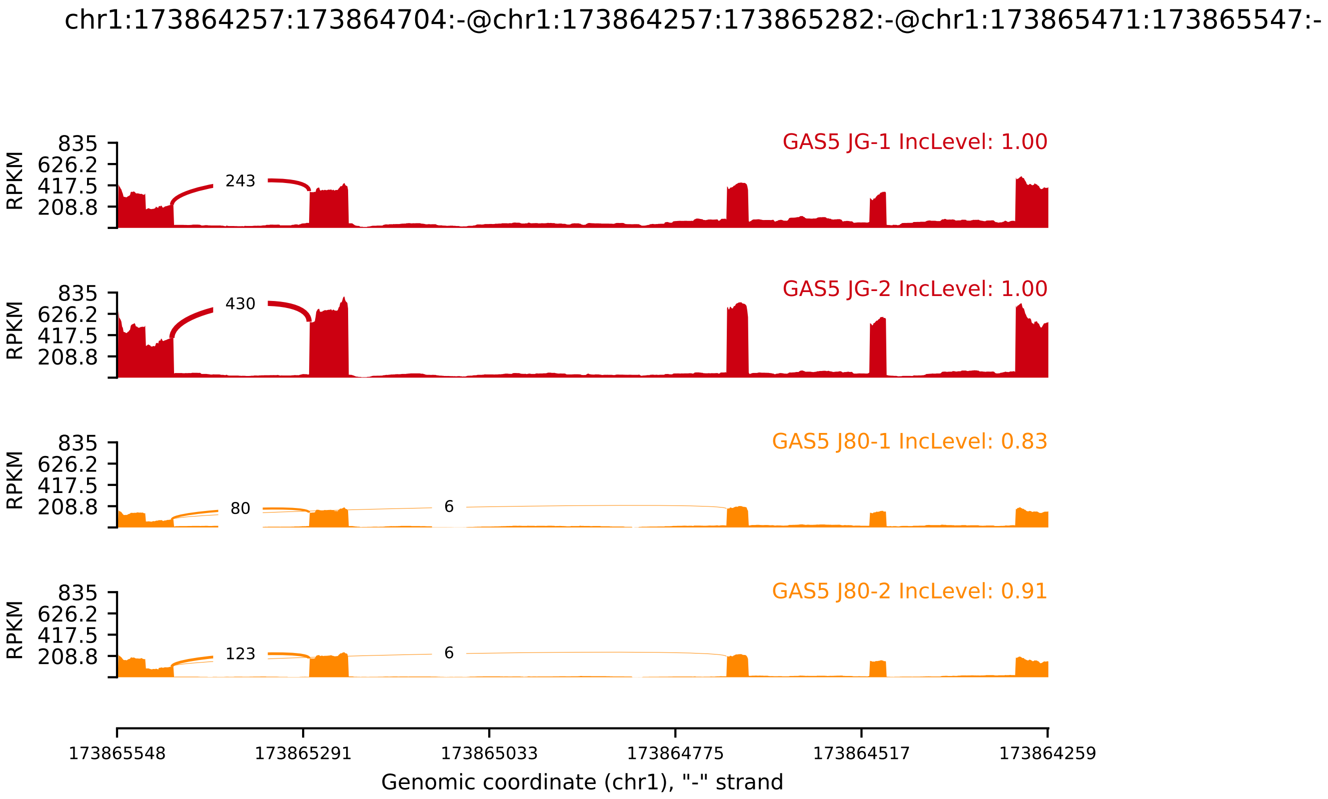

293FT-pX vs 293FT-80-1

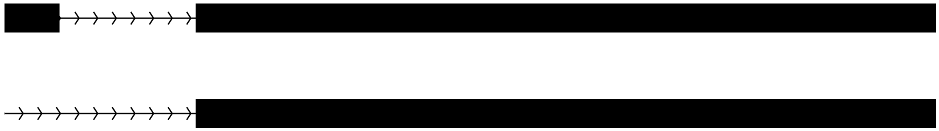

A3SS

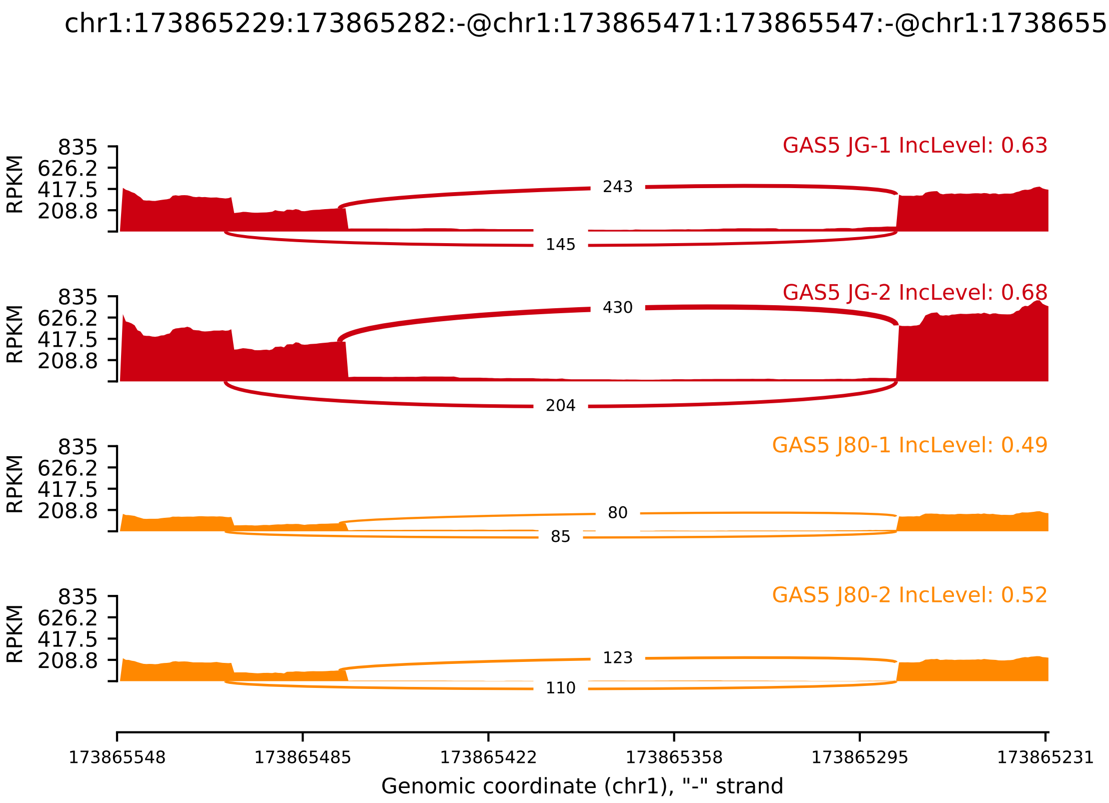

293FT-pX vs 293FT-80-1

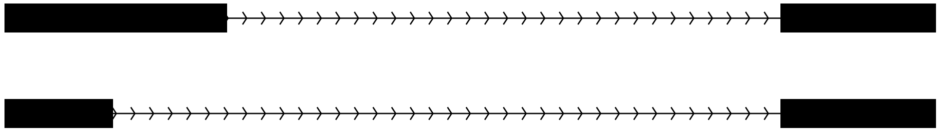

A5SS

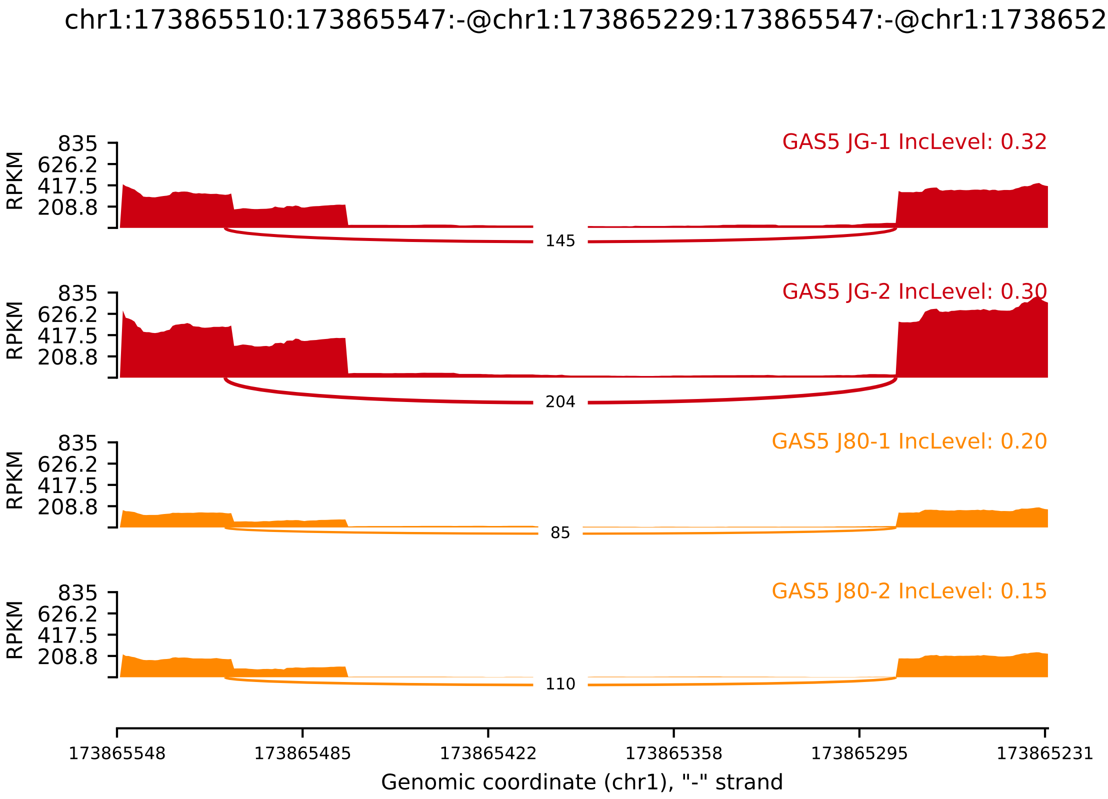

293FT-pX vs 293FT-80-1

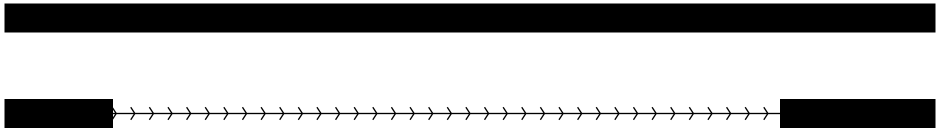

RI
