# Supplementary material for: Are Small Nucleolar RNAs “CRISPRable”? A Report on Box C/D Small Nucleolar RNA Editing in Human Cells
Source: Front Pharmacol. 2019 Nov 4;10:1246. doi: 10.3389/fphar.2019.01246 (PMC6856654; doi:10.3389/fphar.2019.01246)
Supplement: Supplementary file 4 [file Table_1.docx]

**Supplementary Figures**

**
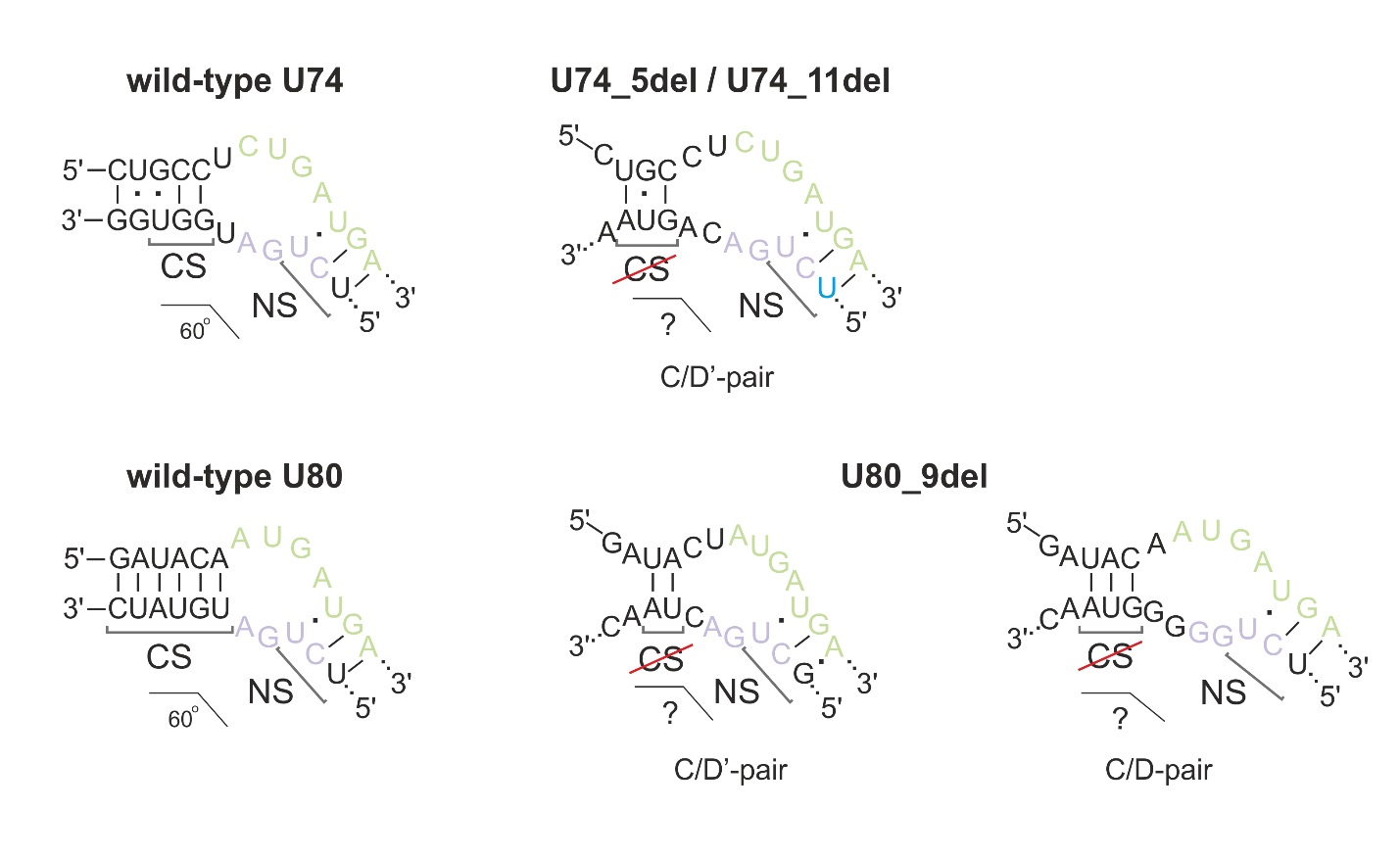
**

**Suppl. Figure 1.** Kink-turn motif structure for wild-type *Gas5* snoRNAs and the proposed K-turn structures for mutant U74 and U80 RNA forms in the obtained monoclones. Nucleotides of the boxes C and D (D’) are colored in green and violet font, respectively. Guide region nucleotide is marked in light blue. CS – canonical stem, NS – non-canonical stem.

**
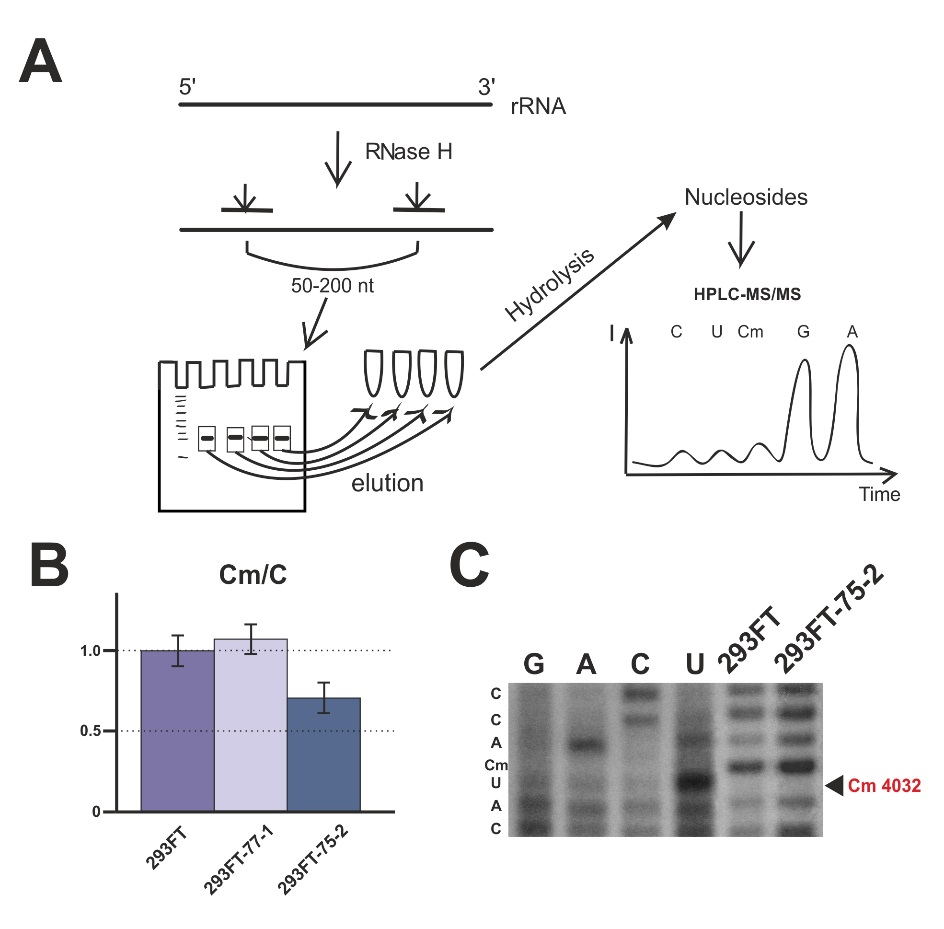
**

**Suppl. Figure 2. (A–C)** Analysis of the 2’-O-methylation status of C4032 28S rRNA in 293FT-75-2 cells using RNase H- and HPLC-MS/MS-based approach (B) and conventional approach based on partial alkaline hydrolysis followed by PCR (C). A – schematic representation of the HPLC-MS/MS-based method. B – 2’-O-methylation level of C4032 28S rRNA in the clones 293FT-75-2 and 293FT-77-1 compared to the control 293FT cells evaluated by HPLC-MS/MS-based method. C – partial alkaline hydrolysis of 28S rRNA followed by RT from total RNA isolated from 293FT and 293FT-75-2 cells.


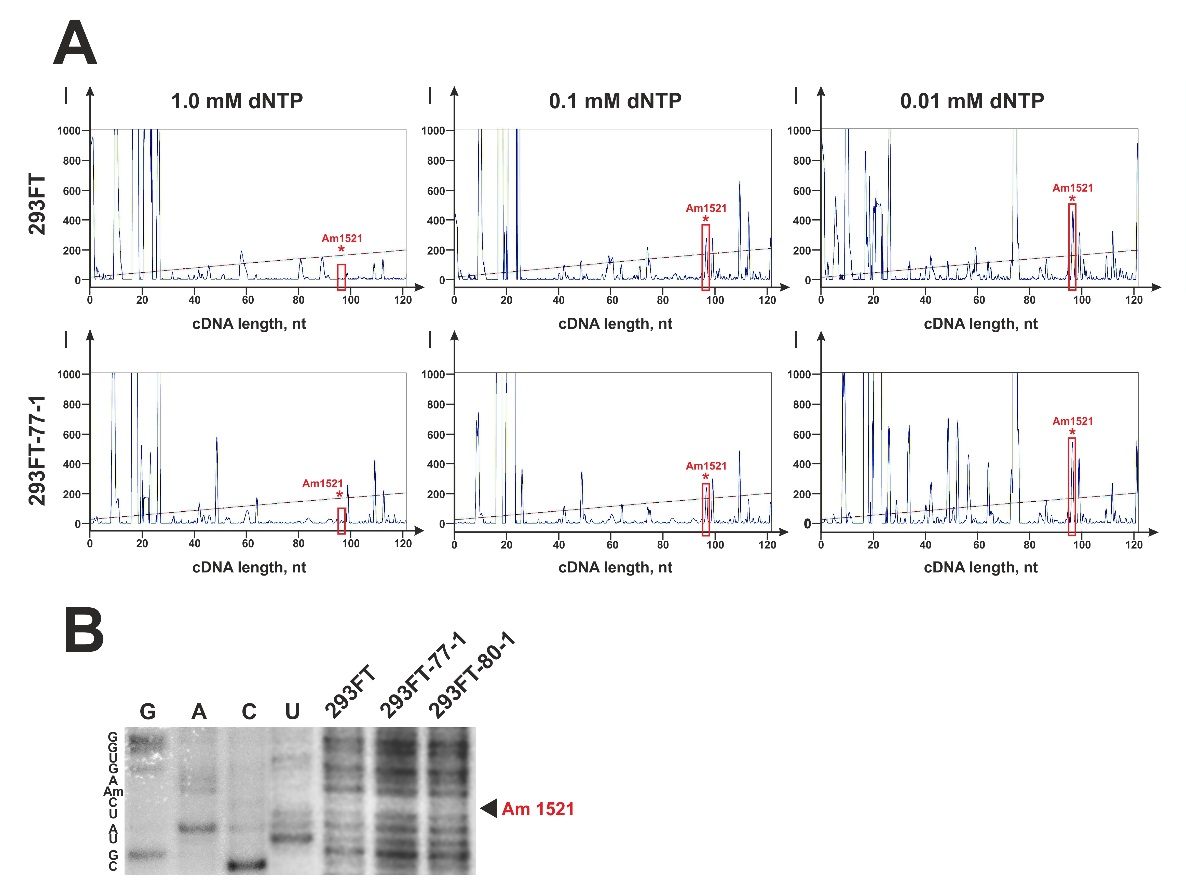


**Suppl. Figure 3. (A–B)** 2’-O-methylation level of C4032 28S rRNA in the clones 293FT-77-1 and 293FT-80-1 compared to the control 293FT cells evaluated by RT (Supplementary Methods) (A) (Filippova et al., 2015) and partial alkaline hydrolysis followed by RT (B). A **–** Upper row: control 293FT cells, lower row: 293FT-77-1 clone. Increase in the level of the corresponding RT termination product (97 nt) at decreased dNTP concentrations indicates the 2’-O-methylated status of A1521 28S rRNA.

Filippova, J. A., Stepanov, G. A., Semenov, D. V., Koval, O. A., Kuligina, E. V., Rabinov, I. V., et al. (2015). Modified Method of rRNA Structure Analysis Reveals Novel Characteristics of Box C/D RNA Analogues. *Acta Naturae* 7, 64–73. Available at: http://www.ncbi.nlm.nih.gov/pubmed/26085946.
