# Supplementary material for: Are Small Nucleolar RNAs “CRISPRable”? A Report on Box C/D Small Nucleolar RNA Editing in Human Cells
Source: Front Pharmacol. 2019 Nov 4;10:1246. doi: 10.3389/fphar.2019.01246 (PMC6856654; doi:10.3389/fphar.2019.01246)
Supplement: Supplementary file 5 [file Table_2.docx]

**Supplementary Methods**

**RT-based method using a fluorescently labeled primer**

Reverse transcription was carried out using primer containing a 5(6)-carboxyfluorescein (FAM) label: 5’-FAM-TCGCCCCTATACCCAGGTCGG-3’ (complementary to the region 1615–1635 in 28S rRNA). The reaction was performed at three dNTP concentrations in parallel: 1mM (optimal dNTP concentration), 0.1 and 0.01 mM (suboptimal dNTP concentrations) as described (Filippova et al., 2015). The products were separated on a BI3100 Genetic Analyzer (Applied Biosystems, Genomics Core Facility, Siberian Branch of the Russian Academy of Sciences). The data were analyzed using Peak Scanner Software version 1.0 (Applied Biosystems, USA).

**Gas5 splicing events analysis**

JunctionSeq (Hartley and Mullikin, 2016) was used to search for differential usage of splice junctions in GAS5 after knockdown of *METTL3*, *METTL14* or *HNRNPC*. The open dataset (GSE56010) was used for analysis. A lot of splice junctions were significantly (adjusted p value % 0.01) differentially used between *METTL14*-versus wild type and also in *METTL3*-versus wild type, as well as *HNRNPC*-versus wild type comparison. According to the picture, we see a significant decrease in the coverage of 5, 7-10 introns after the knockdown of the *METTL3* gene and in that of 3-8 introns after the knockdown of the *HNRNPC* gene.

Filippova, J. A., Stepanov, G. A., Semenov, D. V., Koval, O. A., Kuligina, E. V., Rabinov, I. V., et al. (2015). Modified Method of rRNA Structure Analysis Reveals Novel Characteristics of Box C/D RNA Analogues. *Acta Naturae* 7, 64–73. Available at: http://www.ncbi.nlm.nih.gov/pubmed/26085946.

Hartley, S. W., and Mullikin, J. C. (2016). Detection and visualization of differential splicing in RNA-Seq data with JunctionSeq. *Nucleic Acids Res.*, gkw501. doi:10.1093/nar/gkw501.
