# Supplementary material for: Are Small Nucleolar RNAs “CRISPRable”? A Report on Box C/D Small Nucleolar RNA Editing in Human Cells
Source: Front Pharmacol. 2019 Nov 4;10:1246. doi: 10.3389/fphar.2019.01246 (PMC6856654; doi:10.3389/fphar.2019.01246)
Supplement: Supplementary file 6 [file Table_3.docx]

**Supplementary Table**

Positions of all possible CRISPR/Cas9 cleavage sites in snoRNAs encoded in the introns of *GAS5*.

| **snoRNA** | **Sequence** |
| --- | --- |
| *SNORD74* | 5’-ctgcctc**tgatga**agcctg**^*1^**tgtt**^*2^**g**^*3^**gtagggacat**ctga**cagta**atgatga**atgccaaccgct**c^*4^tga^*5^**tggtgg-3’  3’-gacggag**ac*^6^tact**tcggacac***^7^**aaccatccctgta**gact**gtcat**tactact**tacggttgg***^8^**c**gag*^9^a**ctaccacc-5’ |
| *SNORD*75 | 5’-agcct**gtgatgc**tttaagag***^1^**tagtgga**c*^2^a*^3^ga**agggatttctgaaattctatt***^4^ctga**ggct-3’  3’-tcgga**cac*^5^tacg**aaattctcatcacct**gtct**tccctaaagactttaagataa**gact**ccga-5’ |
| *SNORD*76 | 5’-gccaca**atgatga**cagtttatttgctactc**ttga**gtgctaga**atg*^1^atga**ggatcttaaccaccattatcttaa***^2^ctga**ggc-3’  3’-cggtgt**t*^3^actact**gtcaaataaacgatgag**aact**cacgatct**tactact**cctagaattggtggt***^4^**aat***^5^**agaatt**gact**ccg-5’ |
| *SNORD*77 | 5’-agatact**a*^1^tgatgg**ttgcatagttcag**caga**tttaatc**atgaaga**gatgtactatctgt**ctga**tgtatct-3’  3’-tctatga**tactacc**aacgtatcaagtc**gtct**aaattag**tacttct**ctacatgatagaca**gact**acataga-5’ |
| *SNORD*44 | 5’-cctgg**atgatga**taagcaaatgctgactgaaca***^1^**tgaaggtcttaattagctctaa**ctga**ctaa-3’  3’-ggacc**tactact**attcgtttacgactgacttgtacttccagaattaatcgagatt**gact**gatt-5’ |
| *SNORD*78 | 5’-gtgta**atgatgt**tgatcaaatgtctgac**ctga**aatgagcatgtaga***^1^**caaaggtaaca**ctga**agaa-3’  3’-cacat**tactaca**actagtttacagactg**gact**t***^2^**tactcgtacatctgtttccattgt**gact**tctt-5’ |
| *SNORD*79 | 5’-tactgtta**gtgatga**ttttaaaattaaagc***^1^**a***^2^**gatgggaatctct**ctga**gaaaga***^3^**aaatggagattaatcttaaa**ctga**aacagta-3’  3’-atgacaat**cactact**aaaattttaatttcgtctacccttagaga**ga*^4^c*^5^t**ctttcttttacctctaattagaattt**gact**ttgtcat-5’ |
| *SNORD*80 | 5’-gataca**atgatga**taacatagttcag**caga**ctaacgc**tgatga**gcaatattaagtctttcgctcctat**ctga**tgtatc-3’  3’-ctatgt**tactact**attgtatcaagtc**gtct**gattgcg**actact**cgttataattcagaaagcgaggata**g*^1^act**acatag-5’ |
| *SNORD*47 | 5’-aacca**atgatgt**aatgattctgccaa**atga**aatataatgatatcactgtaaaaccgttccattttgatt***^1^ctga**ggtt-3’  3’-ttggt**tactaca**ttactaagacggtt**ta*^2^ct**ttatattactatagtgacattttggcaag***^3^**gtaaa***^4^**actaa**gact**ccaa-5’ |
| *SNORD*81 | 5’-cagaatac**atgatga**tctcaatccaacttgaactctctca**ctga**ttact**tgatga**caataaaatat**ctga**tattctg-3’  3’-gtcttatg**tactact**agagttaggttga***^1^**acttgagagagt**gact**aatga**actact**gttattttata**gact**ataagac-5’ |

*Conserved elements boxes C, D’, C’ and D sequences are shown in bold and underlined. Positions of CRISPR/Cas9 cleavage sites are denoted by asterisks with numeration.*
